# Release of promoter–proximal paused Pol II in response to histone deacetylase inhibition

**DOI:** 10.1093/nar/gkaa234

**Published:** 2020-04-16

**Authors:** Roshan Vaid, Jiayu Wen, Mattias Mannervik

**Affiliations:** 1 Dept. Molecular Biosciences, The Wenner-Gren Institute, Stockholm University, 10691 Stockholm, Sweden; 2 Dept. Genome Sciences, The John Curtin School of Medical Research, Australian National University, Canberra, ACT 2600, Australia

## Abstract

A correlation between histone acetylation and transcription has been noted for a long time, but little is known about what step(s) in the transcription cycle is influenced by acetylation. We have examined the immediate transcriptional response to histone deacetylase (HDAC) inhibition, and find that release of promoter–proximal paused RNA polymerase II (Pol II) into elongation is stimulated, whereas initiation is not. Although histone acetylation is elevated globally by HDAC inhibition, less than 100 genes respond within 10 min. These genes are highly paused, are strongly associated with the chromatin regulators NURF and Trithorax, display a greater increase in acetylation of the first nucleosomes than other genes, and become transcriptionally activated by HDAC inhibition. Among these rapidly up-regulated genes are HDAC1 (Rpd3) and subunits of HDAC-containing co-repressor complexes, demonstrating feedback regulation upon HDAC inhibition. Our results suggest that histone acetylation stimulates transcription of paused genes by release of Pol II into elongation, and that increased acetylation is not a consequence of their enhanced expression. We propose that HDACs are major regulators of Pol II pausing and that this partly explains the presence of HDACs at active genes.

## INTRODUCTION

A correlation between histone acetylation and transcription was noted for the first time by Vincent Allfrey in the 1960s (1). With the discovery that the transcriptional co-activator Gcn5 is a histone acetyltransferase some 30 years later (2), a more direct link between histone acetylation and gene activation was revealed. At the same time, the transcriptional regulator Rpd3 was shown to be a histone deacetylase (3). The removal of acetyl groups from the epsilon-amino groups of lysine residues is believed to strengthen histone-DNA interactions by increasing the positive charge of histones, and to generate or remove specific docking surfaces for chromatin-binding proteins. This may result in decreased accessibility of nucleosomal DNA to transcription factors and the basal transcription machinery, and histone hypoacetylation is typically associated with transcriptional repression (reviewed in 4). However, chromatin immunoprecipitation studies showed that some histone deacetylases occupy transcriptionally active regions more strongly than silent loci (5). This raises the possibility that histone deacetylation may promote rather than inhibit transcription in some cases (6).

To examine the immediate effects of changes in acetylation to transcription, we have used precision run-on sequencing (PRO-seq) to measure transcription globally in response to the histone deacetylase (HDAC) inhibitor Trischostatin A (TSA). HDACs can be divided into four classes based on sequence homology (reviewed in 7). Metazoan class I HDACs share sequence similarities with the yeast Rpd3 protein, class II with yeast Hda1, class III with yeast Sir2, and class IV, comprised of only HDAC11, shares sequence similarities to both Class I and II HDACs. TSA inhibits class I HDACs and HDAC6 in class II, but not the class IV HDAC and the Sirtuins in class III.

Transcription of mRNA genes involves promoter recognition by RNA Polymerase II (Pol II), followed by initiation, elongation, and termination of transcription (reviewed in 8). In metazoans, Pol II often pauses around 50 bp downstream of the transcription start site (TSS), and release into elongation from promoter–proximal pausing is highly regulated (reviewed in 9). Although Pol II pausing may not serve as an on-off switch of gene expression, pausing is nonetheless important for fine-tuning the transcriptional output of many, if not all genes (9). Despite the strong correlation between histone acetylation and transcription, little is known about which step(s) in the transcription cycle that is affected by histone acetylation. A study in live cells suggested that acetylation of histones stimulates transcriptional elongation without affecting initiation (10). Consistent with this study, we report here that HDAC inhibition does not result in increased initiation, but instead leads to release of promoter–proximal paused Pol II into productive elongation.

## MATERIALS AND METHODS

### Cell culture and drug treatment

We used *Drosophila* S2 cells from DGRC (S2-DRSC stock #006) for most of our experiments. These cells were cultured at 25°C in Schneider's Drosophila Medium (Gibco # 21720024), supplemented with 10% fetal bovine serum (FBS) and 100 units/ml penicillin and 100 μg/ml streptomycin. Human HEK 293 cells were maintained in DMEM (Gibco) supplemented with 10% FBS and 100 Units/ml penicillin and 100 μg/ml streptomycin. Drug treatment was performed for 10 or 30 min on cells resuspended in FBS-free media with either the HDAC inhibitor Trichostatin A (TSA) at 400 nM final concentration, with the transcription inhibitors Flavopiridol (FP) at 300 nM or Triptolide (Trp) at 500 nM final concentration, or in combination, and treatment with the solvent dimethyl sulfoxide (DMSO) was used as a control.

### RNA interference

Double-stranded RNA (dsRNA) targeting Rpd3 and GFP (control) was generated by PCR amplification from cDNA clones using primers containing the T7 RNA polymerase promoter followed by sequences specific for the target genes (shown in Supplementary Table S2). Purified PCR products were used as templates for *in vitro* transcription using the Megascript RNAi kit (Ambion). dsRNA directed against Rpd3 and GFP were 491 and 690 bp respectively. S2 cells (5.2 × 10^6^ cells /treatment) were collected, washed twice and resuspended in 2.5 ml of FBS free medium. 37 nM of dsRNA (Rpd3 or GFP) was added to cells and immediately transferred to a T25 flask and incubated for one hour at 25°C, followed by addition of 2.5 ml medium containing 20% FBS to reach 10% FBS in the total volume. After 2 days the cells were treated again with the same amount of dsRNA in 10 ml of FBS free media and divided into two T75 flasks. An hour later medium containing 20% FBS was added to reach 10% FBS in the total volume and cultured for another 3 days before harvest.

### Precision run-on sequencing (PRO-seq)

Isolation of nuclei and the precision run-on reaction was performed as described previously (11). Since HDAC inhibition could result in a genome-wide transcriptional response, we spiked-in human HEK-293 cells together with the *Drosophila* S2 cells. In brief, cell pellets consisting of 10 million cells treated with DMSO or TSA were resuspended in cold PBS before proceeding to nuclei isolation. At this stage, 0.1 million HEK cells (1% spike-in) also in cold PBS were added to each sample just prior to centrifuging at 700g for 5 min. Nuclei isolation was performed by resuspending cells in buffer A (10 mM Tris–HCl pH 7.5, 300 mM sucrose, 10 mM NaCl, 3 mM CaCl_2_, 2 mM MgCl_2_, 0.1% Triton X, 0.5 mM DTT, protease inhibitors cocktail (Roche), 4 u/ml RNase inhibitor (SUPERaseIN, Ambion) and immediately dounced 25 strokes with a loose pestle. The nuclei pellet was recovered by spinning at 700g for 5 min and washed twice in the same buffer. Finally, isolated nuclei were resuspended in 100 ul buffer D (10 mM Tris–HCl pH 8, 25% glycerol, 5mM MgAc_2_, 0.1 mM EDTA, 5 mM DTT) for every 10 million cells and stored at −80°C. Nuclear run-on was performed exactly as previously described (12).

### ATAC-seq

Crude nuclear extract suitable for ATAC-seq from S2 cells treated with drugs was prepared in ATAC lysis buffer (10 mM Tris pH 7.4, 10 mM NaCl, 3 mM MgCl_2_ 0.1% IGEPAL), followed by tagmentation and library preparation as described earlier (13).

### ChIP-seq and ChIP-qPCR

ChIP experiments were performed as previously described (12), with minor alterations. In brief, drug treated *Drosophila* S2 cells were fixed and quenched. Prior to lysis and sonication, cells were thoroughly washed with cold PBS and then sequentially washed with ChIP A (10 mM HEPES pH 7.6, 10 mM EDTA pH 8.0, 0.5 mM EGTA pH 8.0, 0.25% Triton X100) and ChIP B (10 mM HEPES pH 7.6, 100 mM NaCl, 1 mM EDTA pH 8.0, 0.5 mM EGTA pH 8.0, 0.01% Triton X-100) for 10 min at 4°C followed by resuspension in Sonication buffer (50 mM HEPES, 140 mM NaCl, 1 mM EDTA, 1% Triton, 0.1% sodium deoxycholate, 0.1% SDS, supplemented with proteinase inhibitor tablets, Roche). Chromatin was sheared using a Bioruptor (Diagenode) until an average fragment size range of 200–500 bp was achieved. The solubilized chromatin fraction was cleared by centrifugation and used for immunoprecipitation. Chromatin from HEK-293 cells (corresponding to 1%) prepared in similar buffer conditions was added to serve as spike-in control. Immunoprecipitation with 2 μg of either H3 (Abcam, ab1791), H3K14ac (Abcam, ab52946), H3K27ac (Abcam, ab4729) or RNA Pol II ser2P (Abcam, ab5095) antibodies was carried out at 4°C overnight. A mix of Protein A and G Dynabeads (Invitrogen) blocked with BSA (1 mg/ml) was used to capture the antibody-chromatin complexes. Chromatin and antibody bead complexes were formed during at least 3 hours followed by 10 minute washes with sonication buffer (50 mM HEPES, 140 mM NaCl, 1 mM EDTA, 1% Triton, 0.1% sodium deoxycholate, 0.1% SDS), WashA (as sonication buffer, but with 500 mM NaCl), WashB (20 mM Tris pH 8, 1 mM EDTA, 250 mM LiCl, 0.5% NP-40, 0.5% sodium deoxycholate) and TE. Beads were resuspended in 100 ul TE supplemented with 20 μg/ml RNase A and incubated at 50°C for 30 min. Tris pH 8.0 and SDS were added to these tubes to a final concentration of 50 mM and 0.1% respectively and reversal of the cross-links performed at 68°C for at least 4 h, followed by protein digestion by Proteinase K treatment. Finally, DNA was purified using ChIP DNA Clean & Concentrator™ (Zymo research #D5205) and eluted in 60 μl of DNA elution buffer.

ChIP samples were sequenced on Illumina NextSeq platform at BEA core facility, Stockholm. ChIP-seq libraries were prepared using the NEBNext Ultra II DNA Library Prep Kit (NEB) and single-end (1 × 75 bp) sequenced.

For ChIP-qPCR, 2 μl of DNA was used as template and run in duplicates using 300 nM primers and EvaGreen reagents (Solis BioDyne) on a CFX96 Real-Time system (BioRad). Average Cq was calculated for each ChIP sample and compared to input. To normalize for the background of each individual ChIP, two intergenic sites devoid of known histone modifications and chromatin factors was used. ChIP values of histone modifications were further corrected to the total amount of histone H3. All the primers used in this study are listed in Supplementary Table S2.

### Nuclear RNA isolation and RT-qPCR

We employed exactly the same nuclei isolation protocol as in PRO-seq section (described above) for both *Drosophila* S2 cells and human HEK293 cells. RNA from the isolated nuclei resuspended in Buffer A and solubilized in TRIzol LS reagent (Ambion # 10296010), was extracted using Direct-zol RNA MicroPrep kit (Zymo research # R2060) following the manufacturer's protocol. RNA (1.5 ug) was digested with DNase I (Sigma # AMPD1-1KT) to eliminate genomic DNA. The RNA without any further purification was converted to cDNA using High-Capacity RNA-to-cDNA Kit (ThermoFisher scientific # 4387406) and random primers. RT-qPCR was performed on a CFX96 Real-Time system (BioRad) using gene-specific PCR primers mixed with EvaGreen reagent (Solis BioDyne) and diluted cDNA (7-fold dilution) as template. The PCR primers (Supplementary Table S2) used were designed using Primer3 software, and target intronic regions to capture nascent transcripts. Expression values for each region were normalized to either 28S rRNA, beta-tubulin (*Drosophila*) or ActB (human), using the delta-delta Ct method.

### Western blot

Following drug treatment, nuclear proteins were isolated from S2 cells and crude protein extracts were made from HEK293 cells. S2 cells were washed once with ice cold PBS and re-suspended in cold nuclear lysis buffer (10 mM Tris pH 7.4, 10 mM NaCl, 3 mM MgCl_2_ 0.1% IGEPAL). After 5 min incubation on ice the nuclei were pelleted at 700g for 10 min at 4°C. 1× Laemmli buffer was directly added to the pellet and boiled for 10 min at 95°C. HEK293 cells were washed once with ice cold PBS and resuspended in 1x Laemmli buffer and boiled for 10 min at 95°C.

The samples were electrophoresed on 15% SDS-polyacrylamide gel and transferred on to nitrocellulose membrane. Ponceua S stain was used to confirm transfer before blocking and incubating with primary antibodies diluted in TBST–2% BSA: rabbit monoclonal anti-H3K14ac (1:1000, Abcam, ab52946), rabbit polyclonal anti-H3K27ac (1:1000, Abcam, ab4729), rabbit polyclonal anti-Rpd3 (1:2500, gift from Lori Pile) or mouse monoclonal anti-H3 (1:1000, Millipore 05499) overnight. After washing, the membranes were incubated with fluorophore conjugated secondary antibodies IRDye^®^ 680 RD goat-anti rabbit IgG or IRDye^®^ 800 CW goat-anti mouse IgG at 1:5000 dilution in TBST–2% BSA. Images were acquired on an Infrared Odyssey System (Li-Cor Biosciences) and bands were quantified using IMAGE studio software.

### PRO-seq analysis

After trimming the adaptors and removing the reads that mapped to rRNAs, PRO-seq of DMSO, TSA 10 min, and TSA 30 min in two biological replicates were mapped to the *Drosophila melanogaster* (dm3) genome assembly using BWA with default parameters. To obtain the spike-in reads, we mapped the reads to the human (hg38) genome assembly and counted the reads that only mapped to hg38. We generated PRO-seq coverage tracks, normalized by the spike-ins, with separate strands. Based on PRO-seq reads over gene bodies (500 bp from TSS to 100 bp upstream of TES), we performed differential expression analysis (DE) of TSA 10 min versus DMSO and TSA 30 min versus DMSO using DEseq2 (14). The spike-in reads were used for normalization in the DE. We identified a set of differentially expressed genes with a false discovery rate (FDR) <10% and a set of no-changed genes with an FDR > 50%. The transcript with the longest CDS regions was defined as the canonical transcript for each gene. The genes with an average gene body expression log_2_ transcript per million (TPM) >3 were defined as expressed genes. A pausing index (PI) for canonical transcripts was defined by promoter (−50 to +100 bp) TPM divided by promoter and body (+500 bp to −100 TES) TPM. We obtained chromatin state data and various chromatin regulators and histone modification datasets in S2 cells from the modENCODE project (15). We overlapped the promoter regions (±500 bp around TSS) of PRO-seq body changed sets (up-/down-/no-change) to these datasets. To compare modENCODE ChIP data with different affinities, we standardized the enrichment scores, z-scores, by subtracting the mean and dividing by the standard deviation.

### ChIP-seq analysis

We performed ChIP-seq of DMSO, TSA 10 min and TSA 30 min and Input samples in two replicates with human HEK293 cell chromatin as spike-in, separately for H3K27ac and H3K14ac. We obtained the spike-in reads by mapping the reads to the human (hg38) genome assembly. We mapped ChIP-seq reads to the *Drosophila melanogaster* (dm3) genome assembly using Bowtie2 with default parameters after adaptor trimming by Trimmomatic (16). The uniquely mapped reads with a mapping quality MAPQ >20 were used for further analysis. We generated coverage tracks for ChIP-seq samples, which were normalized to spike-in and Input samples. We performed peak calling of ChIP-seq reads against Input reads by Genrich peak caller (https://github.com/jsh58/Genrich), which took the replicate consistency into account. As we aim to call differential peaks downstream, we here set a relatively relaxed peak-calling threshold (*P*-value < 0.01) to obtain candidate peak regions. The peaks that overlapped with a *Drosophila* blacklist were removed (17). To perform differential ChIP-seq analysis between TSA versus DMSO on the called peaks, we first merged the peaks of the compared pairs by ‘mergePeaks’ function in Homer2 package and set a maximum distance to ‘-d given’ which requires the peak regions to overlap. ChIP-seq reads on the merged peak regions were normalized by spike-in and differential strengths of the peaks were identified by DESeq2 with a FDR <10%. The ChIP-seq heatmap and meta-gene plots of the normalized coverage tracks centered at the TSS or peak summits were generated by deepTools2 and custom scripts (18).

### ATAC-seq analysis

The ATAC-seq samples of DMSO, TSA 10 min and TSA 30 min in two biological replicates were mapped to the *Drosophila melanogaster* (dm3) genome assembly using Bowtie2 with default parameters after adaptor trimming by Trimmomatic. The high quality and uniquely mapped reads (MAPQ ≥ 20) were used for further analysis. We performed peak calling of ATAC-seq accessible regions separately for DMSO, TSA 10 min and TSA 30 min by Genrich peak caller with parameter setting (Genrich -t -o -f -r -j -y -d 100 -q 0.05 -e chrM -v). ATAC-seq differential accessibility analysis between TSA and DMSO on the ATAC-seq peaks was performed with the DEseq2 package. Nucleosome positioning analysis was performed by NucleoATAC (19).

Sequencing library size statistics are included in Supplementary Table S3.

## RESULTS

### HDAC inhibition causes instantaneous transcriptional up-regulation of a few genes

**Figure 1.**
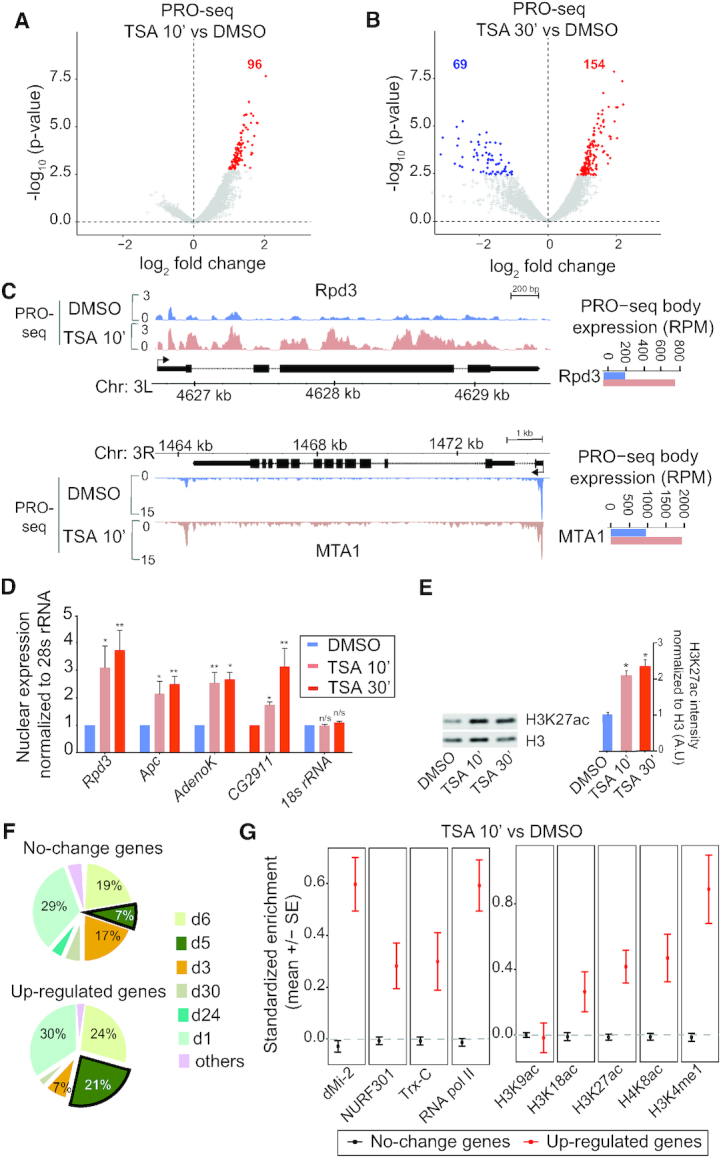
Histone deacetylase inhibition results in rapid transcriptional up-regulation of genes associated with a distinct chromatin state. (A and B) PRO-seq gene body (500 bp from TSS – 100 bp upstream of TES) log_2_ fold change (x-axis) versus -log_10_*P*-values (y-axis) in *Drosophila* S2 cells treated with the HDAC inhibitor TSA versus DMSO control for 10 min (**A**) or 30 min (**B**). (**C**) PRO-seq signal in DMSO control and TSA-treated S2 cells shown for the *Rpd3* and *MTA1* loci. (**D**) RT-qPCR with nuclear RNA isolated from S2 cells treated with DMSO or TSA for 10 or 30 min normalized against 28S rRNA. *n* = 4. Error bars represent SEM and significant differences between control and TSA-treated cells (two-tailed paired *t*-test) are indicated by asterisks, * *P*< 0.05, ** *P*< 0.01. (**E**) Western blot showing H3K27 acetylation and total H3 in DMSO and TSA-treated cells. The H3K27ac signal was normalized to total H3 and error bars represent SEM, *n* = 4. Significant differences between control and TSA-treated cells (two-tailed paired *t*-test) are indicated by asterisks, * *P*< 0.05. (**F**) Association of promoter regions (±500 bp around TSS) of 96 up-regulated genes within 10 min of TSA and genes that do not change their expression with modENCODE chromatin states. (**G**) Average standardized enrichment scores (z-scores) of dMi-2 (NuRD complex), NURF301 (NURF complex), Trithorax (Trx-C), Pol II and histone modifications in unchanged and 96 up-regulated gene promoters.

In order to elucidate the direct consequence of an altered histone acetylation state on transcription, we treated *Drosophila* S2 cells with the HDAC inhibitor Trichostatin A (TSA) or DMSO as control for 10 or 30 min and performed precision run-on sequencing (PRO-seq). This method captures transcriptionally engaged polymerases at base-pair resolution (20), and circumvents the issue of RNA stability that is associated with conventional RNA-seq experiments. To normalize the PRO-seq data we spiked-in untreated human HEK293 cells at the nuclei isolation step of the procedure (see Methods). Based on PRO-seq read distribution over gene bodies (500 bp from TSS to 100 bp upstream of TES) in the samples, 6315 genes with an average gene body log_2_ transcripts per million (TPM) >3 were defined as expressed genes (Supplementary Figure S1A). Using DEseq2 and a 10% false discovery rate (FDR < 0.1) to identify differential PRO-seq gene body reads between control and TSA-treated samples, we found that 96 genes were significantly up-regulated already after 10 min of HDAC inhibition, but that no genes were down-regulated at this time-point (Figure 1A and Supplementary Table S1). This gives support to the notion that HDAC catalytic activity represses transcription. After 30 min of TSA treatment, more up-regulated genes (154), but also a number of down-regulated genes (69) were identified (Figure 1B). It is possible that the down-regulated genes are indirect HDAC targets, being affected by repressors that are up-regulated after 10 min. Consistent with this view, we found that several components of the Sin3/Rpd3 and NuRD co-repressor complexes, including Rpd3 (HDAC1), Sin3A, SAP130 and MTA1, as well as sequence-specific repressors such as Ken and Brinker were more highly expressed in 10 min TSA-treated than in control cells (Figure 1C and Supplementary Table S1). This also suggests that there is a rapid feed-back regulation in TSA-treated cells, with HDAC inhibition resulting in stronger HDAC expression. Using intronic primers in RT-qPCR from nuclear RNA, we confirmed the up-regulation of *Rpd3* and three more genes after 10 min of TSA treatment (Figure 1D). The majority of genes that are up-regulated after 10 min are also more strongly expressed after 30 min of TSA treatment than in control cells (Figure 1D and Supplementary Figure S1B). Interestingly, all up-regulated genes are already expressed in S2 cells before TSA treatment (Supplementary Figure S1C), suggesting that HDACs are involved in maintaining a moderate expression level of these genes. As expected, histone acetylation was increased globally by HDAC inhibition (Figure 1E). Western blots showed that both H3K27ac and H3K14ac increased approximately 1.5- to 2-fold after 10 min of TSA treatment (Figure 1E and Supplementary Figure S1D). We found that this rapid response is evolutionarily conserved, as HDAC1 and the p27 CDK inhibitor were up-regulated after 10 min of TSA-treatment also in human HEK293 cells (Supplementary Figure S1E), along with elevated levels of global H3K27ac (Supplementary Figure S1F). Together these results suggest that the direct effects of lysine deacetylation on transcription is restricted to repression and is not involved in gene activation.

To evaluate if there are any special features associated with the gene promoters that respond quickly to HDAC inhibition, we investigated what fraction were associated with different chromatin states identified in S2 cells by the modENCODE project (15). We found that state d5, enriched in H3K4me3, H3K9ac and H3K27ac, is more common in the up-regulated genes compared to genes that are not affected after 10 min (Figure 1F). To gain further insight into the features of these quickly responding genes we examined enrichment levels of various chromatin regulators and histone modifications. We found that these genes have higher levels of dMi-2, a subunit of the NuRD complex that also contains Rpd3/HDAC1 (reviewed in 21), compared to genes that do not change in expression (Figure 1G). Other factors such as the nucleosome remodeler NURF (reviewed in 22), and the H3K4me1 methylase Trithorax (Trx) (reviewed in 23) were also enriched compared to non-affected genes, along with H3K4me1 and histone marks associated with active genes such as H4K8ac, H3K18ac and H3K27ac (Figure 1G and Supplementary Figure S2). However, H3K9ac levels were comparable between up-regulated and non-affected genes, indicating that up-regulated genes are associated with a unique chromatin state. Interestingly, Pol II occupancy was also higher at these genes in untreated cells (Figure 1G). We searched for DNA motifs that are enriched at the promoters of these genes, and compared core promoter motifs between up-regulated and non-affected genes (Supplementary Figure S1G and H). This did not reveal any striking sequence feature in the up-regulated genes. A gene ontology analysis showed that these genes are involved in development and cell signaling (Supplementary Figure S1I). We conclude that a subset of developmental and signaling genes respond quickly to HDAC inhibition and that these are marked with certain chromatin regulators, active histone modifications and high levels of Pol II.

### HDAC inhibition results in release from promoter–proximal pausing

**Figure 2.**
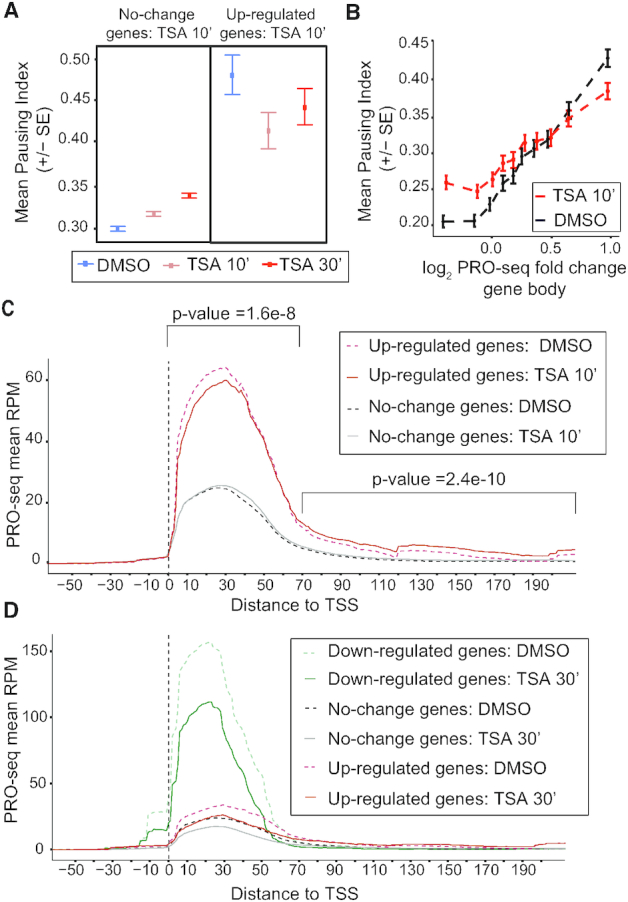
HDAC inhibition up-regulates promoter–proximal paused genes by releasing Pol II into elongation. (**A**) Mean pausing index (PRO-seq reads from promoter–proximal, −50 to +100-bp region, versus gene body, +500 bp to TES) for unchanged genes (left) and up-regulated genes (right) after 10 min of TSA treatment in DMSO, TSA 10 min and TSA 30 min-treated S2 cells. (**B**) Correlation between PRO-seq gene body fold change and pausing index in DMSO and TSA 10 min treated cells. (**C**) Metagene PRO-seq profiles for up-regulated and no change genes after 10 min of TSA. Significant differences between control and TSA-treated cells (two-tailed paired t-test) for the 96 up-regulated genes are indicated. (**D**) Metagene PRO-seq profiles for down-regulated, up-regulated and no change genes after 30 min of TSA.

Since the genes that responded to HDAC inhibition have high Pol II occupancy and are moderately expressed (Figure 1G, Supplementary Figure S1C), we speculated that these genes are paused. To address this we directly measured the pausing index from the PRO-seq signal in the −50 bp to +100 bp region compared to the gene body, +500 from the TSS to −100 bp of the TES. In line with our expectations, genes that are up-regulated after 10 min have a higher pausing index than un-affected genes before TSA addition (Figure 2A, DMSO treatment). After 10 min of TSA, the pausing index of the up-regulated genes decreases, whereas it slightly increases in genes whose expression does not change (Figure 2A). After 30 min of TSA, there is a further increase in the pausing index of non-affected genes, but they are still less paused than the up-regulated genes (Figure 2A). We plotted the pausing index versus the PRO-seq expression change, which showed that the most highly paused genes have the strongest change in expression (Figure 2B). After 10 min of TSA, the pausing index decreases for strongly up-regulated genes but increases for weakly up-regulated genes and genes that do not change expression or are slightly down-regulated (Figure 2B). We conclude that the most highly paused genes in S2 cells are the ones that respond to TSA already after 10 min.

The decrease in pausing index observed at the up-regulated genes after TSA treatment could be caused by a decrease in Pol II recruitment, by an increase in release into productive elongation, or a combination of the two. A metagene plot showed that genes that are up-regulated within 10 mins of HDAC inhibition on average have a small reduction of Pol II at the pause site (+1 – 70 bp, *P* = 1.6E–8) and increased PRO-seq read densities in the gene bodies (+70 – 200 bp, *P* = 2E–10, Figure 2C). The difference in PRO-seq reads can also be seen in a plot of the ratio of PRO-seq reads before and after TSA treatment (Supplementary Figure S3A). Importantly, this shows that up-regulated genes do not increase their expression due to increased initiation. Instead, paused Pol II is more efficiently released into elongation at these genes after TSA treatment. Consistent with this finding, we observed increased Ser2 phosphorylated Pol II at up-regulated genes after 10 min of TSA by ChIP-qPCR (Supplementary Figure S3B).

Genes that do not change expression have on average a slightly increased PRO-seq read density at 40–60 bp downstream of the TSS after 10 min of TSA (Figure 2C), consistent with their increased pausing index (Figure 2A and B). However, 30 min after TSA addition, non-affected genes as well as up-regulated and down-regulated genes all have less PRO-seq reads on average in the first 80 bp of the gene (Figure 2D and Supplementary Figure S3C). Although some genes have higher PRO-seq read densities in the promoter–proximal region after 30 min of TSA-treatment (e.g. Rpd3 and MTA1, Supplementary Figure S3D), we conclude that TSA treatment does not result in increased initiation at the majority of genes. Instead, less Pol II is on average associated with the pause site in response to TSA. Despite this, there are more genes that increase their expression than those that have decreased expression.

Taken together, these data show that highly paused genes respond quickly to HDAC inhibition by releasing Pol II into productive elongation more efficiently than in untreated cells.

### Chromatin accessibility changes more slowly than transcription in response to HDAC inhibition

**Figure 3.**
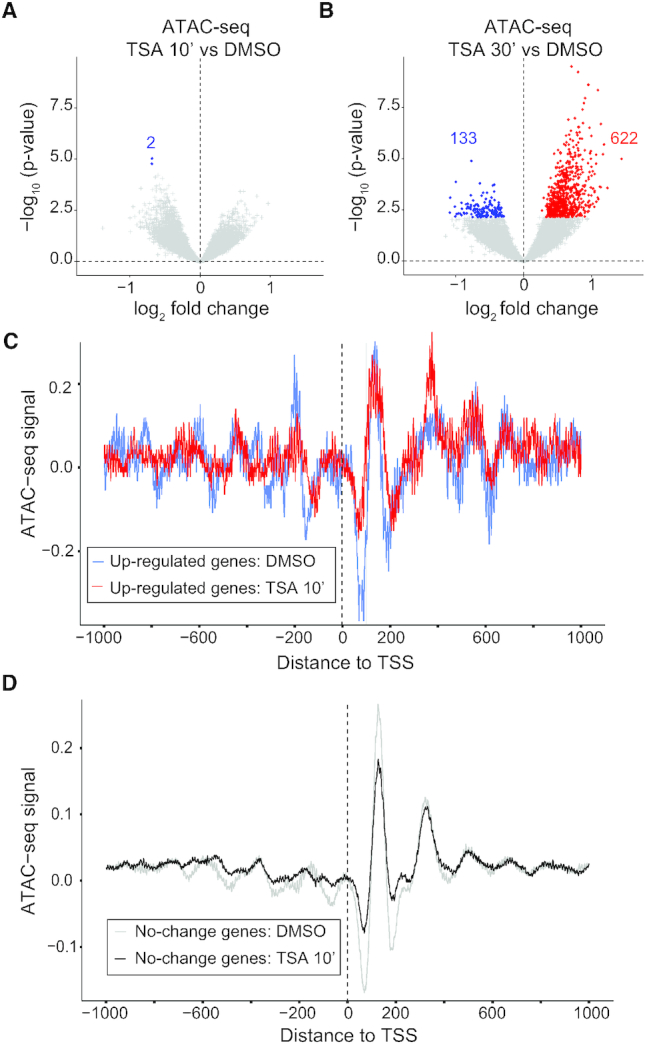
Chromatin accessibility and nucleosome positioning is similar in control and HDAC inhibitor-treated cells. (A and B) Volcano plots of ATAC-seq log_2_ fold change versus –log_10_*P*-values on the ATAC-seq peak regions in S2 cells treated with TSA versus DMSO control for 10 min (**A**) or 30 min (**B**). (C and D) Nucleosome position ±1 kb around the TSS from ATAC-seq data in DMSO or 10 min TSA-treated cells for 96 up-regulated genes (**C**) and non-affected genes (**D**) after 10 min of TSA.

One function for promoter–proximal pausing is to maintain an open chromatin state at promoters by competing with nucleosomes (24). To determine whether the change in pausing index has an effect on chromatin accessibility we performed ATAC-seq on control and TSA-treated cells. We found no regions with increased accessibility after 10 min of HDAC inhibition, and only two regions with significantly decreased accessibility (Figure 3A). Consistent with these results, we did not detect changes in H3 occupancy by ChIP-qPCR at the regions tested (Supplementary Figure S4A). After 30 min of TSA treatment, 795 regions showed increased accessibility and 443 decreased accessibility compared to control cells (Figure 3B). Some of these regions with changed accessibility are located within 2 kb of a promoter, and out of 337 genes with altered accessibility after 30 min, eight genes had increased their transcription already by 10 min of TSA treatment. This shows that transcription was elevated before chromatin accessibility changed at these eight genes. We also used the ATAC-seq data to examine if nucleosome positions were changed by TSA. We found no difference in the positioning of the first four nucleosomes after 10 min of TSA treatment, neither at the 96 up-regulated genes (Figure 3C), nor at the genes whose expression was unaffected (Figure 3D). After 30 min of TSA, there was also no global change in nucleosome positioning (Supplementary Figure S4B–D). Our results suggest that transcriptional alterations precede changes to chromatin accessibility and that the increase in histone acetylation by itself has only minor effects on the accessible regions.

### Transcriptional changes correlate with altered histone acetylation levels

**Figure 4.**
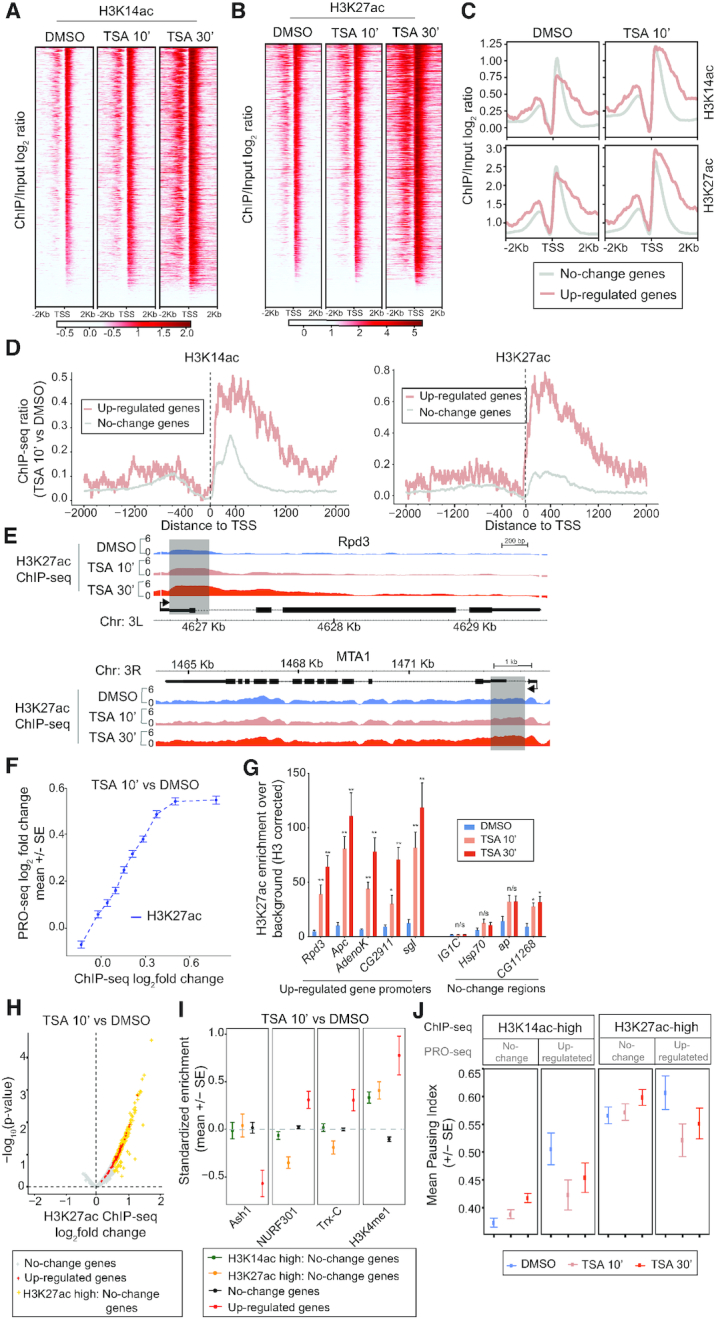
Gain in histone acetylation after HDAC inhibition correlates with transcriptional output and is greatest in the first 2 kb of up-regulated genes. (A and B) Heatmaps of H3K14ac (**A**) and H3K27ac (**B**) ±2 kb around the TSS in DMSO, TSA 10 min and TSA 30 min-treated cells sorted on the level of acetylation. (**C**) Metagene plots of H3K14ac (top) and H3K27ac (bottom) ±2 kb around the TSS for 96 up-regulated and non-affected genes in DMSO and 10 min TSA-treated cells. (**D**) H3K14ac (left) and H3K27ac (right) log_2_ fold-change ±2 kb around the TSS at up-regulated and unaffected genes. (**E**) H3K27ac log_2_ ChIP-seq signal over input at the *Rpd3* and *MTA1* loci in DMSO, TSA 10 min and TSA 30 min-treated cells. Regions with a strong increase after 10 min are shaded. (**F**) Correlation between H3K27ac promoter (±1 kb around TSS) fold change and PRO-seq gene body fold change. (**G**) H3K27ac ChIP-qPCR at up-regulated gene promoters and control loci from S2 cells treated with DMSO or TSA for 10 or 30 min normalized against H3 occupancy. *n* = 3. Error bars represent SEM and significant differences between control and TSA-treated cells (two-tailed paired t-test) are indicated by asterisks, * *P*< 0.05, ** *P*< 0.01. (**H**) H3K27ac ChIP-seq peak (±1 kb around TSS) fold changes after 10 min TSA. The peak with the largest change per gene was plotted. The 96 up-regulated genes are labeled red, and PRO-seq gene body unchanged (FDR > 0.5) with >1.5-fold H3K27ac increase are labeled orange. (**I**) Average standardized enrichment scores (*z*-scores) of Ash1, NURF301, Trithorax (Trx-C) and H3K4me1 in gene promoters with >1.5-fold increase in H3K14ac or H3K27ac without a transcriptional change, in PRO-seq unchanged with less acetylation changes, and in 96 up-regulated gene promoters. (**J**) Mean pausing index (based on PRO-seq) for genes with >1.5-fold increase in H3K14ac (left) or H3K27ac (right) where PRO-seq gene body reads do not change or are increased after 10 min of TSA treatment in DMSO, TSA 10 min and TSA 30 min-treated S2 cells.

To investigate how altered transcription correlates with histone acetylation changes, we performed ChIP-seq and ChIP-qPCR experiments using H3K14ac (associated with active promoters) as well as H3K27ac (marking active promoters and enhancers) antibodies. We performed ChIP-seq experiments for both of these histone modifications in TSA-treated S2 cells with human HEK293 cell chromatin as spike-in. Consistent with a global increase in histone acetylation after TSA addition as determined by Western blot (Figure 1D), elevated H3K14ac and H3K27ac was also detected by ChIP-seq after spike-in normalization as can be seen in heat-maps centered around the TSS (Figure 4A and B). The levels of these histone modifications were increased by 10 min and were even higher after 30 min of HDAC inhibition (Figure 4A and B). Although average H3K27 acetylation at the 96 up-regulated genes was higher than for unchanged genes in untreated cells according to modENCODE data (Figure 1G), the level of acetylation at the +1 nucleosome was equivalent or slightly lower in DMSO control cells (Figure 4C). Instead, higher acetylation levels were observed upstream and further downstream of the TSS.

Interestingly, the increase in histone acetylation after 10 min of TSA was greater in the 96 genes that were transcriptionally up-regulated than in unaffected genes (Figure 4C and D). The largest increase was observed downstream of the TSS, at positions corresponding to the +1 and +2 nucleosomes for H3K27ac and for the first 3–4 nucleosomes for H3K14ac (Figure 4D). This is also evident at the individual gene level (Figure 4E). Plotting the change in histone acetylation versus the change in transcription revealed a strong correlation between the two (Figure 4F and Supplementary Figure S5A), indicating that increased acetylation may be responsible for the elevated transcription.

We also examined histone acetylation changes at enhancer regions, defined as CBP peaks located more than 100 bp from the TSS (Supplementary Figure S5B-C). Up-regulated genes had on average higher H3K27ac at their enhancers compared to non-changed genes before TSA treatment, and the average increase in acetylation was greater at up-regulated genes than at un-affected genes (Supplementary Figure S5D). Taken together, our results suggest that genes that respond transcriptionally to HDAC inhibition have a larger increase in their histone acetylation state in both enhancer and promoter–proximal regions than genes whose expression is not affected, and that this is proportional to the amount of up-regulation.

After 30 min of TSA we found that up-regulated genes had the largest increase in histone acetylation, followed by unaffected genes, and that down-regulated genes showed the smallest increase in histone acetylation (Supplementary Figure S5E). A strong correlation between histone acetylation and transcription was observed also at this time point, with promoter regions with the biggest increase in ChIP-seq signals being the most strongly up-regulated (Supplementary Figure S5F). Consistent with these results, ChIP-qPCR showed that H3K27ac increased at several up-regulated genes, including *Rpd3, Apc, AdenoK, CG2911* and *sgl* after 10 min of TSA-treatment, whereas the change at the non-affected genes *Hsp70, ap* and *CG11268* and some intergenic regions was either smaller or not significant (Figure 4G). At 30 min after TSA addition, the up-regulated genes had even higher H3K27ac also by ChIP-qPCR (Figure 4G). We conclude that histone acetylation increases the most in transcriptionally up-regulated genes after TSA treatment.

To confirm that these findings are specific to HDAC inhibition, we knocked down Rpd3 by RNAi. This resulted in increased global H3K27ac, a strong increase in acetylation over TSA-target genes, and compensatory *Rpd3* transcription (Supplementary Figure S5G–I).

Despite the strong correlation between changes to transcription and histone acetylation, we identified 268 genes associated with increased H3K27 acetylation and 688 genes with increased H3K14ac >1.5-fold whose transcription does not change significantly after 10 min of TSA (Figure 4H, Supplementary Figure S5J). To see if we could separate features associated with increased acetylation from features associated with transcriptional up-regulation, we compared modENCODE data sets to these regions of greatest increase in H3K27ac and H3K14ac after TSA treatment that are not associated with expression changes. Although many factors are enriched both at more acetylated regions and transcriptional up-regulated genes compared to non-affected genes, NURF-301, Trx and H3K4me1 occupy the up-regulated genes more strongly than regions with increased acetylation without expression changes (Figure 4I and Supplementary Figure S6). By contrast, the H3K36 di-methylase Ash1 (23), is depleted from up-regulated gene promoters (Figure 4I). Taken together, our results demonstrate a strong correlation between transcription and histone acetylation, but show that increased histone acetylation alone cannot predict which genes will become transcriptionally up-regulated.

Since both the amount of increased acetylation and the pausing index correlate with the transcriptional changes, we selected all genes associated with at least a 1.5-fold increase in acetylation and divided them into non-changed and up-regulated genes, and plotted the mean pausing index in DMSO, TSA 10 min and TSA 30 min-treated cells (Figure 4J). This shows that among genes with the greatest increase in acetylation, up-regulated genes are on average more paused before TSA than genes that do not change their expression. We conclude that genes that respond to HDAC inhibition gain histone acetylation to a larger extent than most genes, are more highly paused than genes that gain acetylation to the same extent, and are more strongly associated with NURF, Trx and H3K4me1 than other genes.

### Transcription is not needed for increased histone acetylation after HDAC inhibition

**Figure 5.**
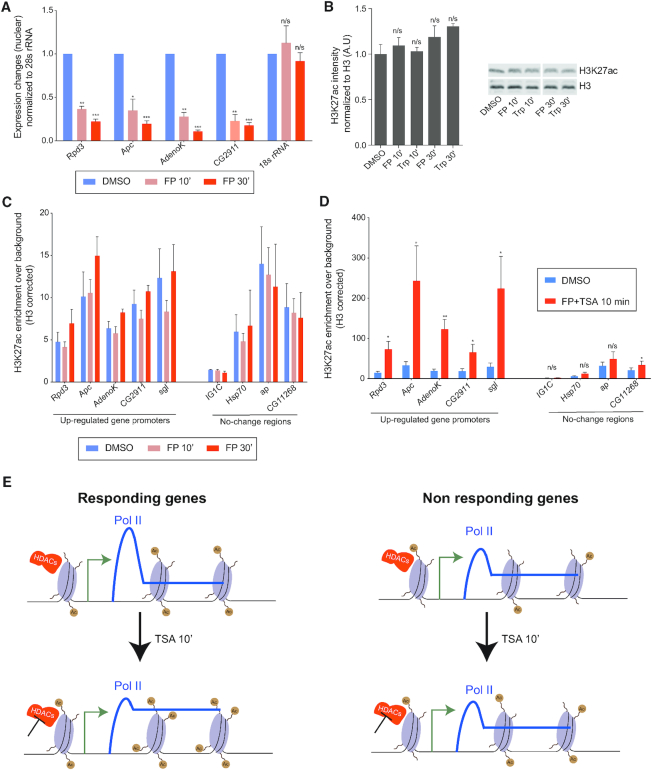
Inhibiting transcription does not prevent an increase in histone acetylation upon HDAC inhibition. (**A**) RT-qPCR with nuclear RNA isolated from S2 cells treated with DMSO control or Flavopiridol (FP) for 10 or 30 min normalized against 28S rRNA. *n* = 3. (**B**) Quantification of H3K27ac Western blot after Flavopiridol (FP) or Triptolide (Trp) treatment, *n* = 2. A representative blot is shown to the right. (**C**) H3K27ac ChIP-qPCR at up-regulated gene promoters and control loci from S2 cells treated with DMSO or FP for 10 or 30 min normalized against H3 occupancy. *n* = 3. (**D**) H3K27ac ChIP-qPCR at up-regulated gene promoters and control loci from S2 cells treated with DMSO or TSA and FP for 10 min normalized against H3 occupancy. *n* = 3. (**E**) Schematic model of the transcriptional response to HDAC inhibition. Error bars represent SEM and significant differences between control and treated cells (two-tailed paired *t*-test) are indicated by asterisks, * *P*< 0.05, ** *P*< 0.01, *** *P <* 0.001.

To investigate if the strong increase in histone acetylation that was observed at transcriptionally up-regulated genes was a consequence of elevated transcription, we treated S2 cells with the transcription inhibitors Triptolide (Trp) or Flavopiridol (FP). Trp inhibits TFIIH and thereby prevents transcription initiation, whereas FP is a PTEF-b inhibitor that blocks release from promoter–proximal pausing (reviewed in 25). By using intronic primers in RT-qPCR from nuclear RNA we confirmed that 10 min of FP treatment resulted in fewer nascent transcripts from the *Rpd3, Apc, AdenoK* and *CG2911* genes, whereas Trp had a significant effect at all tested genes only after 30 min (Figure 5A, Supplementary Figure S7A). We then used Western blot to investigate if transcription inhibition resulted in global changes to histone acetylation, but did not detect any significant difference in cells treated with Trp or FP for 10 or 30 min (Figure 5B). To examine if transcription inhibition resulted in changes to histone acetylation at the *Rpd3, Apc, AdenoK, CG2911* and *sgl* loci, we performed H3K27ac ChIP-qPCR. We found that acetylation was not significantly affected at these loci, nor at control loci that are not sensitive to TSA, by either FP or Trp addition (Figure 5C, Supplementary Figure S7B). Together, these results suggest that momentarily inhibiting transcription has little effect on histone acetylation.

To investigate if increased H3K27ac occurs in TSA-treated cells when transcription is inhibited, we treated cells with both FP and TSA for 10 min and performed ChIP-qPCR (Figure 5D). Increased histone acetylation was detected at all five gene loci in these cells, but was less strong or non-significant at control loci that do not respond to HDAC inhibition (Figure 5D). We conclude that TSA treatment results in elevated levels of histone acetylation even in the absence of transcription, which suggests that increased histone acetylation precedes and may cause transcription activation of these genes.

## DISCUSSION

Despite the strong correlation between histone acetylation and transcription, little is known about the mechanisms by which transcription is stimulated by acetylation. We show here that release of paused Pol II into elongation is the predominant mechanism by which acetylation increases transcription. Several studies have suggested that histone acetylation results in open chromatin that is more accessible to DNA-binding proteins (26–28). Although our results show that several regions display increased accessibility after 30 min of HDAC inhibition as assessed by ATAC-seq, there are only small changes after 10 min despite a global increase in histone acetylation. This indicates that many changes to chromatin accessibility may be a consequence of altered protein binding and active chromatin remodeling, rather than a direct effect of histone acetylation on accessibility. At both 10 and 30 min after HDAC inhibition, the amount of transcriptionally engaged Pol II in the promoter–proximal region is not increased at most genes, arguing against an effect of acetylation on Pol II initiation. It remains possible that Pol II recruitment is enhanced without an increase in initiation. Instead, we find that Pol II is released into productive elongation more efficiently after HDAC inhibition. This is consistent with a study in live mammalian cells that demonstrated an effect of acetylation on transcription elongation rather than initiation (10).

A recent study showed that Sirt6 restrains release from promoter–proximal pausing by preventing release of the negative elongation factor NELF, and by impeding recruitment of positive elongation factors such as BRD4 and the P-TEFb kinase (29). However, TSA does not inhibit Sirt6 activity, suggesting that class I or II HDACs possess a similar ability to prevent transcription elongation. Another study suggested that H3K9 acetylation releases paused Pol II into transcription elongation by recruiting the super elongation complex to chromatin (30). The SEC contains P-TEFb that phosphorylates the elongation factors NELF, DSIF and the Pol II C-terminal domain (CTD) to release Pol II from pausing (reviewed in 31). It is possible that this is how HDAC inhibition causes release into elongation. Not only histones but also several elongation factors are acetylated (e.g. 32), so HDACs could restrain release from pausing by deacetylating these proteins as well. Another possibility is that increased acetylation of the first nucleosomes facilitates Pol II elongation, consistent with in vitro studies showing that acetylation stimulates transcription (33–35). The first nucleosome represents a stronger barrier to elongation than subsequent nucleosomes (reviewed in 36), suggesting that histone acetylation may be particularly important close to the TSS. We have previously reported that inhibiting the histone acetyltransferase CBP causes an accumulation of Pol II upstream of the +1 nucleosome concomitant with decreased acetylation (12), indicating that acetylation of nucleosomes influences Pol II elongation. Whether acetylation only influences release of paused Pol II into elongation or if it also affects the elongation rate remains to be shown.

Although histone acetylation is increased globally already by 10 min of HDAC inhibition, few genes respond transcriptionally. Interestingly, all of these 96 genes are activated, suggesting that the only direct effect HDACs have on transcription is repression. This indicates that the strong HDAC occupancy that is often observed at active genes functions to restrict gene expression. Similarly, although promoter–proximal pausing restrains Pol II from entering productive elongation, it is positively correlated with gene expression (9). This suggests that it may function to tune transcription in response to cellular state. Of note, the genes that become up-regulated upon HDAC inhibition are highly paused, and there is a striking correlation between pausing index and the amount of up-regulation. We suggest that HDACs may be key regulators of pausing, possibly by controlling the recruitment of P-TEFb, and that this may explain why HDACs are found at many active genes.

Interestingly, up-regulated genes gain acetylation to a larger extent than other genes when HDACs are inhibited, presumably because they are highly occupied by HDACs. Consistent with this idea, dMi-2, a subunit of the HDAC-containing NURD complex is more strongly associated with up-regulated than with non-affected genes. Of note, the larger gain in acetylation is mainly confined to the first 2 kb downstream of the TSS. This indicates that HDACs are associated with promoters and downstream positions close to the TSS where they function to restrain Pol II elongation. However, there are also genes that gain acetylation to the same extent without being up-regulated. This shows that histone acetylation is not sufficient for transcription activation at many genes. Perhaps other proteins are being acetylated that cause gene activation. It is also possible that up-regulated genes are associated with features that allow them to respond to increased acetylation. We found that the chromatin remodeler NURF and the Trithorax histone methyltransferase occupy up-regulated genes more strongly than genes that gain acetylation without an increase in transcription. One possibility is that NURF and Trithorax restrict P-TEFb recruitment to these genes making them highly paused, and that increased acetylation results in enhanced P-TEFb recruitment causing release into productive elongation.

Whether histone acetylation is a consequence of transcription or causes an increase in transcription is debated (37). A recent study demonstrated that global acetylation is largely dependent on transcription (38). By contrast, our results show that within a short time frame of transcription inhibition there is no global effect on histone acetylation, nor does histone acetylation change in the presence of transcription inhibitors at genes that are sensitive to HDAC inhibition. However, histone acetylation is increased at these genes when both HDACs and transcription is inhibited. This shows that the strong increase in histone acetylation at genes up-regulated upon HDAC inhibition is not due to their elevated expression. Instead it suggests that histone acetylation is causing increased transcription of these genes.

Among the up-regulated genes are cell cycle regulators such as the p21/p27 CDK inhibitor Dacapo, which is also a major target for HDACs in mammalian cells and is believed to contribute to the cell cycle arrest induced by HDAC inhibitors in tumor cells (reviewed in 39). This indicates that some of the direct targets are evolutionarily conserved and that cells of different origin adapt to changes in acetylation in similar ways. Indeed, HDAC1 and p27 expression was up-regulated already after 10 min also in human HEK293 cells. Intriguingly, many of the up-regulated genes are components of HDAC-containing co-repressor complexes, including HDAC1/Rpd3 itself. This shows that cells have evolved a feed-back mechanism to sense reduced HDAC activity. We suggest that this feed-back mechanism involves release of promoter–proximal paused Pol II at these genes, and that increased acetylation of the first few nucleosomes allows for a more efficient release of Pol II into productive elongation. Given that factors conserved among metazoans are involved in this process, we believe that rapid release of paused Pol II in response to increased acetylation occurs also in mammals and other animals, but not in yeast cells that lack Pol II pausing.

In conclusion, we have found that HDAC inhibition causes rapid transcriptional up-regulation of highly paused genes by releasing promoter–proximal paused Pol II into productive elongation (Figure 5E). Our results suggest that increased acetylation of the first nucleosomes facilitates this release of paused Pol II into elongation and provide a mechanism by which histone acetylation stimulates transcription. The pausing index is altered for most genes after HDAC inhibition, suggesting that HDACs may be key regulators of pausing. This may partly explain the enigmatic presence of HDACs at active genes, since many active genes are paused. The use of an HDAC inhibitor in combination with a nuclear run-on assay allowed us to identify genes that respond within minutes to increased acetylation, and to distinguish between direct and indirect effects of HDAC inhibition. Since we only found up-regulated genes at the earliest time point, we conclude that the direct effect of HDAC catalytic activity on transcription is limited to repression. We also discovered transcriptional feedback regulation of HDAC expression and conclude that release of Pol II from pausing is the mechanism used by cells to compensate for lowered HDAC activity. The identification of features associated with direct target genes could guide efforts to reduce toxicity and resistance to HDAC inhibitors used clinically in the treatment of various cancers.

## DATA AVAILABILITY

The datasets generated and analysed during the current study are available in the GEO repository, accession GSE141871. https://www.ncbi.nlm.nih.gov/geo/query/acc.cgi?acc=GSE141871. UCSC genome browser session: https://genome.ucsc.edu/s/Jeanwen/dm3_TSA.

## Supplementary Material

gkaa234_Supplemental_Files
