# Supplementary material for: Release of promoter–proximal paused Pol II in response to histone deacetylase inhibition
Source: Nucleic Acids Res. 2020 Apr 16;48(9):4877–90. doi: 10.1093/nar/gkaa234 (PMC7229826; doi:10.1093/nar/gkaa234)
Supplement: gkaa234_Supplemental_Files [file gkaa234_supplemental_files.zip › Supplemental_Data.pdf]

Supplemental Data to

Release of promoter-proximal paused Pol II in response to histone deacetylase inhibition

Roshan Vaid, Jiayu Wen and Mattias Mannervik

Supplemental Figure S1. Related to Figure 1.

(**A**) Histograms depicting log_2 transcript per million (TPM) distribution (x-axis) of PRO-seq reads for all genes in S2 cells treated with either DMSO or TSA for the times indicated. Biological duplicates are denoted as rep1 and rep2. (**B**) Venn diagram showing the overlap of up-regulated genes (based on PRO-seq gene body) between 10 min and 30 min TSA treatment. (**C**) Histograms showing PRO-seq gene expression profiles (log_2 TPM distribution, x-axis) for no change genes (left) and genes up-regulated after 10 min of TSA (right) in DMSO treated S2 cells. (**D**) Western blot quantification measuring changes in H3K14ac levels normalized to H3 after TSA treatment, n=2, error bars represent SEM. Representative blot probed with H3K14ac and H3 antibodies is shown below. (**E**) RT-qPCR with nuclear RNA isolated from human HEK293 cells treated with DMSO or TSA for 10 min normalized against Actin B mRNA. n=3. Error bars represent SEM and significant differences between control and TSA-treated cells (two-tailed paired t-test) are indicated by asterisks, * $p<0.05$. (**F**) Western blot quantification measuring changes in H3K27ac levels in HEK293 cells normalized to H3 after TSA treatment, n=2, error bars represent SEM. Representative blot probed with H3K27ac and H3 antibodies is shown. (**G**) De novo motifs identified by MEME-ChIP (FDR 0.1) at upstream 200 bp – downstream 100 bp around TSS for the up-regulated genes after 10 min of TSA treatment. (**H**) Frequency of core promoter motifs identified with the ElemeNT tool (1), at all expressed genes, genes up-regulated after 10 min, 30 min or down-regulated after 30 min of TSA. (**I**) Gene ontology analysis (biological process, left) and Molecular Signatures Database (MSigDB, right) enrichment for the 96 up-regulated genes after 10 min TSA treatment.

Supplemental Figure S2. Related to Figure 1

Average standardized enrichment scores (z-scores) of various chromatin regulators and histone modifications between no change genes and up-regulated genes after 10 min TSA treatment. Data is from the modENCODE project (2), and some factors are present at very few genes (large error bars) or are absent from the up-regulated genes.

Supplemental Figure S3. Related to Figure 2

(**A**) Metagene plots showing PRO-seq ratios (TSA 10 min/DMSO) samples -50bp to +200 bp around TSS at up regulated genes and no change genes. (**B**) Ser2-phosphorylated Pol II ChIP-qPCR at up-regulated gene promoters and control loci from S2 cells treated with DMSO or TSA for 10 min. n=2. Error bars represent SEM and significant differences between control and TSA-treated cells (two-tailed unpaired t-test) are indicated by asterisks, * $p<0.05$. (**C**) Metagene plots showing PRO-seq ratios (TSA 30 min/DMSO) samples -50bp to +200 bp around TSS at up regulated genes, no change genes and down regulated genes. (**D**) Examples of up-regulated genes after TSA treatment. PRO-seq signal over the *Rpd3* and *MTA1* genes, in DMSO control, TSA 10 and 30 min. Tracks shown are PRO-seq signal normalized to spike-in.

Supplemental Figure S4. Related to Figure 3

(**A**) Histone H3 ChIP-qPCR at up-regulated gene promoters and control loci from S2 cells treated with DMSO or TSA for 10 or 30 min. n=2. Error bars represent SEM. No significant differences between control and TSA-treated cells were observed. (**B-D**)

Nucleosome position +/- 1 kb around TSS, measured by ATAC-seq for up-regulated genes (**B**) no change genes (**C**) and down-regulated genes (**D**) in DMSO or 30 min TSA treated S2 cells.

Supplemental Figure S5. Related to Figure 4

(**A**) Correlation between H3K14ac promoter (+/- 1 kb around TSS) fold change and PRO-seq gene body fold change in TSA 10 min versus DMSO. (**B**) Heatmaps showing enrichment of enhancer H3K27ac ChIP-seq signal +/- 1 kb around CBP peak summits (3), located more than 100 bp from the TSS. (**C**) Metagene plots of H3K27ac ChIP-seq +/- 1 kb around CBP peak summits located more than 100 bp from the TSS for 96 up-regulated genes and no change genes in DMSO, TSA 10 and 30 min. (**D**) H3K27ac ChIP-seq $\log_2$ fold change (TSA 10 min/DMSO) at enhancers for 96 up-regulated genes and no change genes. (**E**) H3K14ac (left) and H3K27ac (right) $\log_2$ fold change (TSA 30 min/DMSO) +/- 2 kb around TSS for up-regulated, down-regulated and no change genes. (**F**) Correlation between H3K14ac (left) H3K27ac (right) ChIP-seq fold change (TSA 30 min/DMSO) and PRO-seq gene body fold change (TSA 30 min/DMSO). (**G**) Western blots of Rpd3, H3K27ac and H3 in Rpd3 RNAi and control GFP RNAi-treated cells. Quantification is shown to the right. (**H**) H3K27ac ChIP-qPCR at TSA-regulated gene promoters and control loci from control GFP RNAi and Rpd3 RNAi-treated S2 cells. n=2. Error bars represent SEM. (**I**) RT-qPCR with nuclear RNA isolated from GFP or Rpd3 RNAi-treated S2 cells normalized against beta-tubulin mRNA. n=2. (**J**) H3K14ac ChIP-seq peak (+/- 1 kb around TSS) fold changes after 10 min TSA. The peak with the largest change per gene was plotted. The 96 up-regulated genes are labeled red, and PRO-seq gene body unchanged (FDR > 0.5) with >1.5-fold H3K14ac increase are labeled orange.

Supplemental Figure S6. Related to Figure 4

Average standardized enrichment scores (z-scores) of various chromatin regulators and histone modifications between four groups of genes - High H3K14ac (>1.5-fold) no change (PRO-seq) genes, High H3K27ac (>1.5-fold) no change (PRO-seq) genes, no change genes without increased histone acetylation, and up-regulated genes after 10 min TSA treatment.

Supplemental Figure S7. Related to Figure 5

(**A**) RT-qPCR with nuclear RNA isolated from S2 cells treated with DMSO control or Triptolide (Trp) for 10 or 30 min normalized against 28S rRNA. n=3 (**B**) H3K27ac ChIP-qPCR at up-regulated gene promoters and control loci from S2 cells treated with DMSO or Trp for 10 or 30 min normalized against H3 occupancy. n=3. Error bars represent SEM and significant differences between control and Trp-treated cells (two-tailed paired t-test) are indicated by asterisks, * $p<0.05$, ** $p<0.01$.

References

1. Sloutskin, A., Danino, Y.M., Orenstein, Y., Zehavi, Y., Doniger, T., Shamir, R. and Juven-Gershon, T. (2015) ElemeNT: a computational tool for detecting core promoter elements. *Transcription*, **6**, 41-50.
2. Roy, S., Ernst, J., Kharchenko, P.V., Kheradpour, P., Negre, N., Eaton, M.L., Landolin, J.M., Bristow, C.A., Ma, L., Lin, M.F. *et al.* (2010) Identification of functional elements and regulatory circuits by Drosophila modENCODE. *Science*, **330**, 1787-1797.
3. Philip, P., Boija, A., Vaid, R., Churcher, A.M., Meyers, D.J., Cole, P.A., Mannervik, M. and Stenberg, P. (2015) CBP binding outside of promoters and enhancers in Drosophila melanogaster. *Epigenetics Chromatin*, **8**, 48.

Figure S1

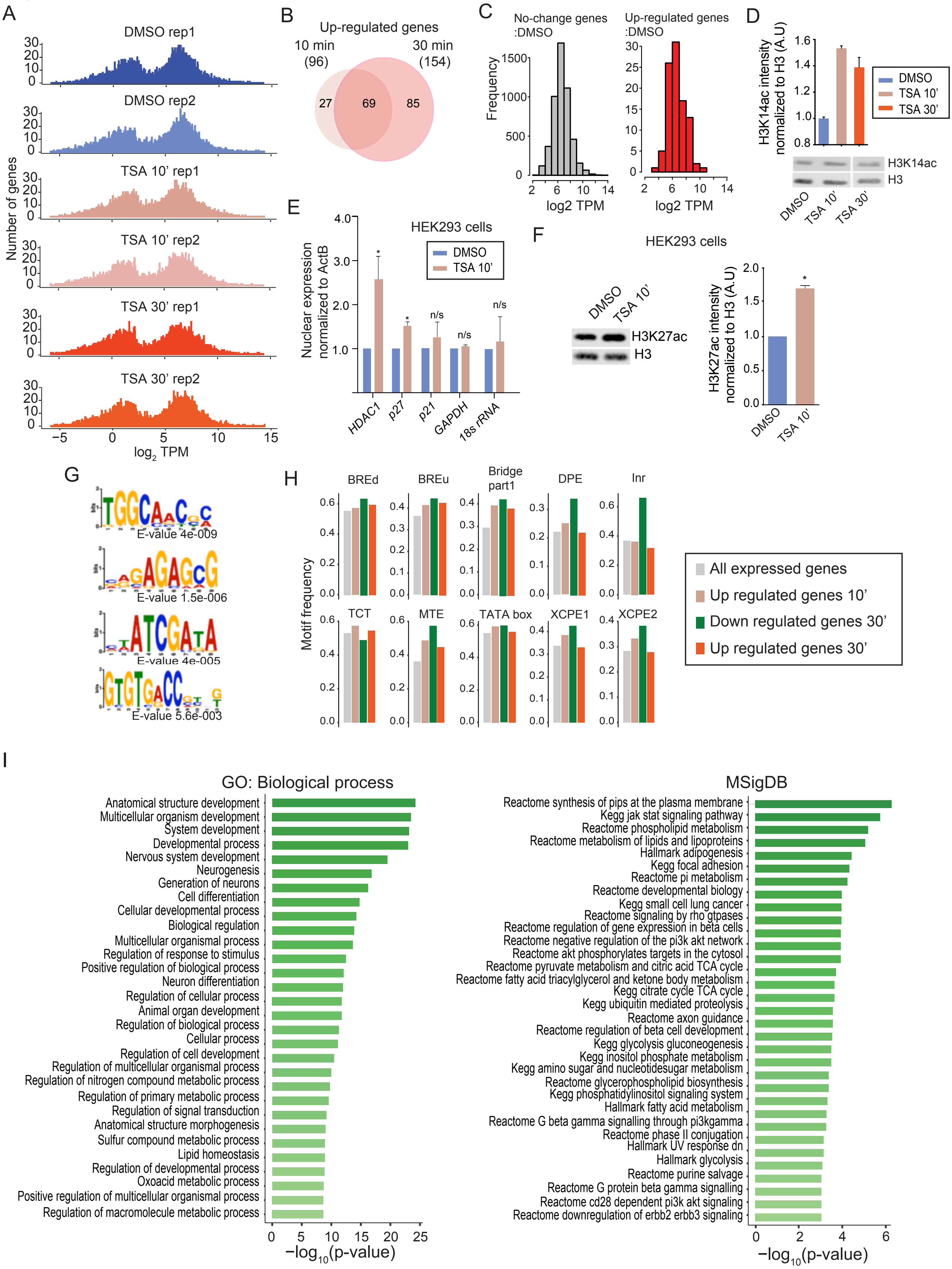
A
DMSO rep1
DMSO rep2
TSA 10’ rep1
TSA 10’ rep2
TSA 30’ rep1
TSA 30’ rep2
Number of genes
log2 TPM
B
Up-regulated genes
10 min (96)
30 min (154)
27
69
85
C
No-change genes :DMSO
Up-regulated genes :DMSO
Frequency
log2 TPM
D
H3K14ac intensity normalized to H3 (A.U)
DMSO
TSA 10’
TSA 30’
H3K14ac
H3
E
HEK293 cells
Nuclear expression normalized to ActB
DMSO
TSA 10’
n/s
HDAC1
p27
p21
GAPDH
18s rRNA
F
HEK293 cells
DMSO
TSA 10’
H3K27ac
H3
H3K27ac intensity normalized to H3 (A.U)
G
E-value 4e-009
E-value 1.5e-006
E-value 4e-005
E-value 5.6e-003
H
BREd
BREu
Bridge part1
DPE
Inr
TCT
MTE
TATA box
XCPE1
XCPE2
Motif frequency
All expressed genes
Up regulated genes 10’
Down regulated genes 30’
Up regulated genes 30’
I
GO: Biological process
Anatomical structure development
Multicellular organism development
System development
Developmental process
Nervous system development
Neurogenesis
Generation of neurons
Cell differentiation
Cellular developmental process
Biological regulation
Multicellular organismal process
Regulation of response to stimulus
Positive regulation of biological process
Neuron differentiation
Regulation of cellular process
Animal organ development
Regulation of biological process
Cellular process
Regulation of cell development
Regulation of multicellular organismal process
Regulation of nitrogen compound metabolic process
Regulation of primary metabolic process
Regulation of signal transduction
Anatomical structure morphogenesis
Sulfur compound metabolic process
Lipid homeostasis
Regulation of developmental process
Oxoacid metabolic process
Positive regulation of multicellular organismal process
Regulation of macromolecule metabolic process
−log10(p-value)
MSigDB
Reactome synthesis of pips at the plasma membrane
Kegg jak stat signaling pathway
Reactome phospholipid metabolism
Reactome metabolism of lipids and lipoproteins
Hallmark adipogenesis
Kegg focal adhesion
Reactome pi metabolism
Reactome developmental biology
Kegg small cell lung cancer
Reactome signaling by rho gtpases
Reactome regulation of gene expression in beta cells
Reactome negative regulation of the pi3k akt network
Reactome akt phosphorylates targets in the cytosol
Reactome pyruvate metabolism and citric acid TCA cycle
Reactome fatty acid triacylglycerol and ketone body metabolism
Kegg citrate cycle TCA cycle
Kegg ubiquitin mediated proteolysis
Reactome axon guidance
Reactome regulation of beta cell development
Kegg glycolysis gluconeogenesis
Kegg inositol phosphate metabolism
Kegg amino sugar and nucleotidesugar metabolism
Reactome glycerophospholipid biosynthesis
Kegg phosphatidylinositol signaling system
Hallmark fatty acid metabolism
Reactome G beta gamma signalling through pi3kgamma
Reactome phase II conjugation
Hallmark UV response dn
Hallmark glycolysis
Reactome purine salvage
Reactome G protein beta gamma signalling
Reactome cd28 dependent pi3k akt signaling
Reactome downregulation of erbb2 erbb3 signaling
−log10(p-value)

Figure S2

TSA 10’ vs DMSO

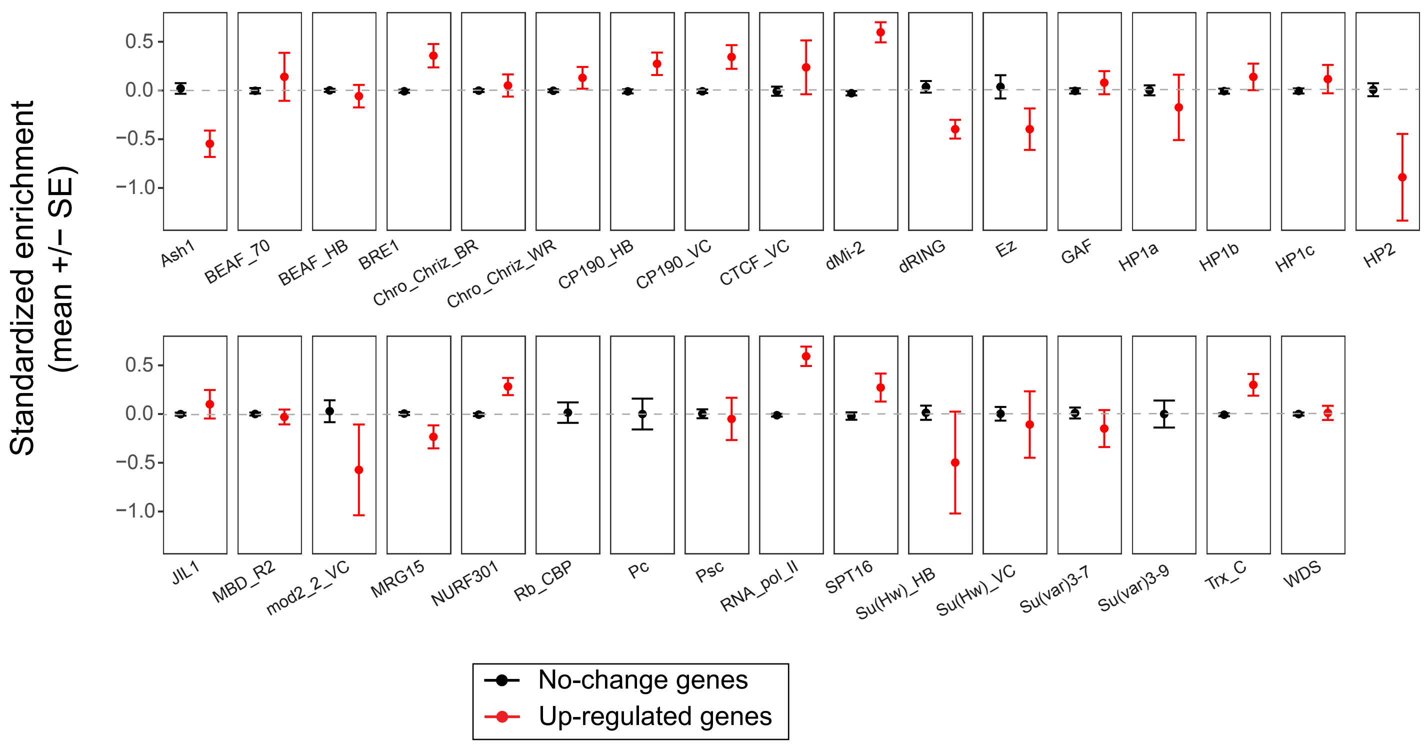

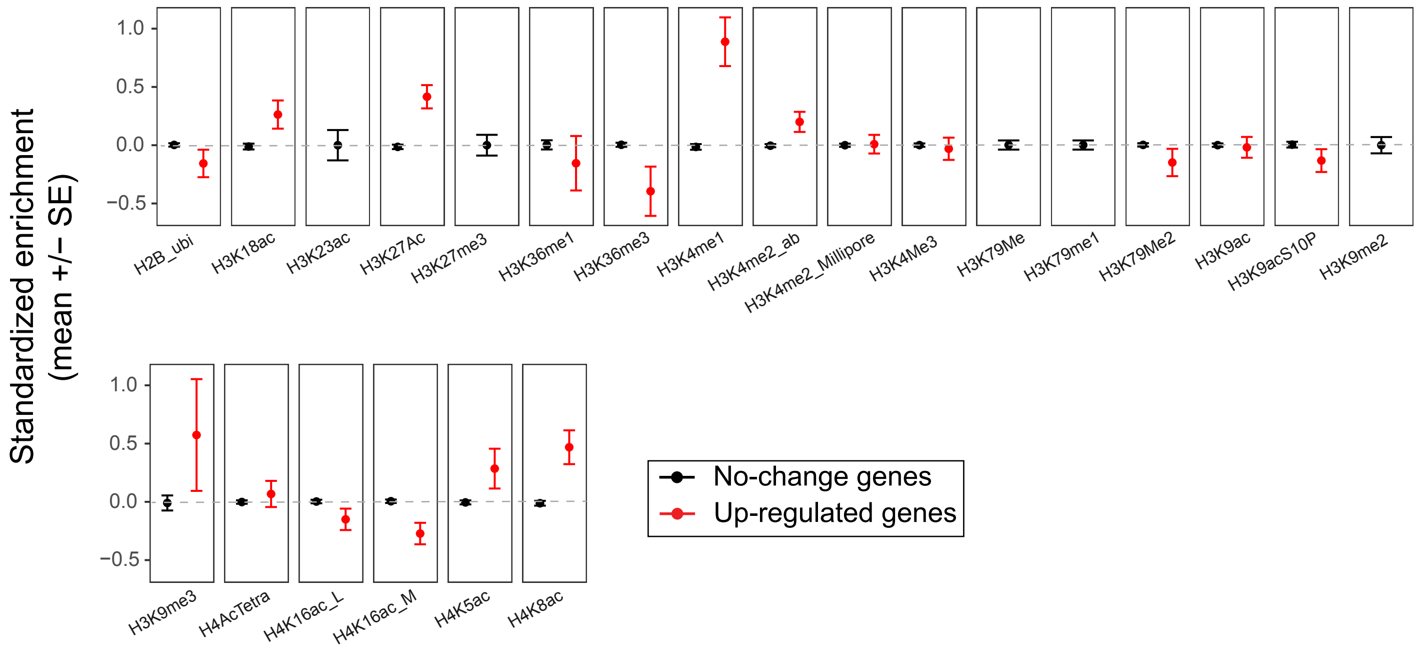

Figure S3

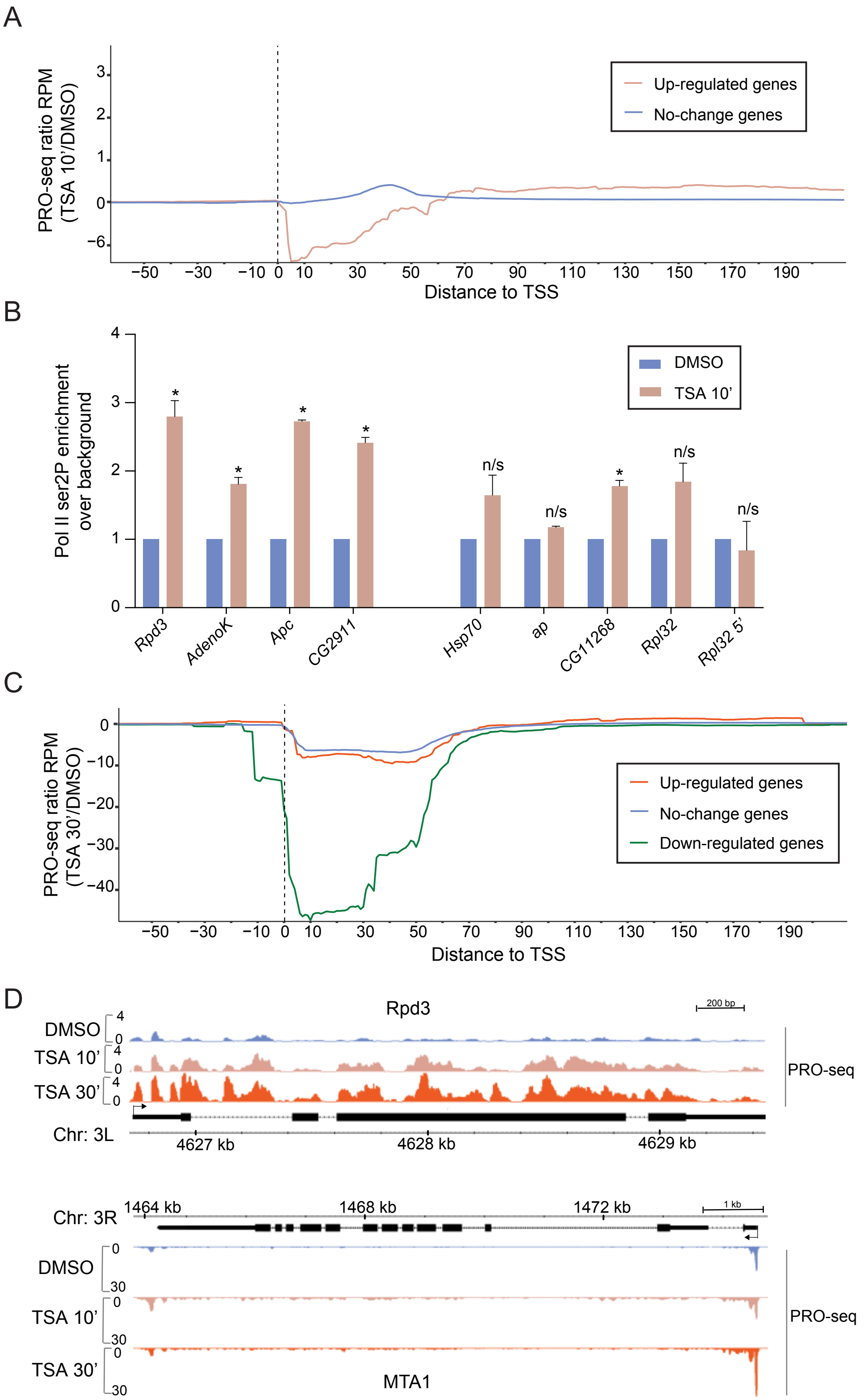
A
Up-regulated genes
No-change genes
PRO-seq ratio RPM
(TSA 10'/DMSO)
Distance to TSS
B
DMSO
TSA 10'
Pol II ser2P enrichment
over background
*
n/s
Rpd3
AdenoK
Apc
CG2911
Hsp70
ap
CG11268
Rpl32
Rpl32 5'
C
Up-regulated genes
No-change genes
Down-regulated genes
PRO-seq ratio RPM
(TSA 30'/DMSO)
Distance to TSS
D
Rpd3
200 bp
DMSO
TSA 10'
TSA 30'
PRO-seq
Chr: 3L
4627 kb
4628 kb
4629 kb
Chr: 3R
1464 kb
1468 kb
1472 kb
1 kb
MTA1

Figure S4

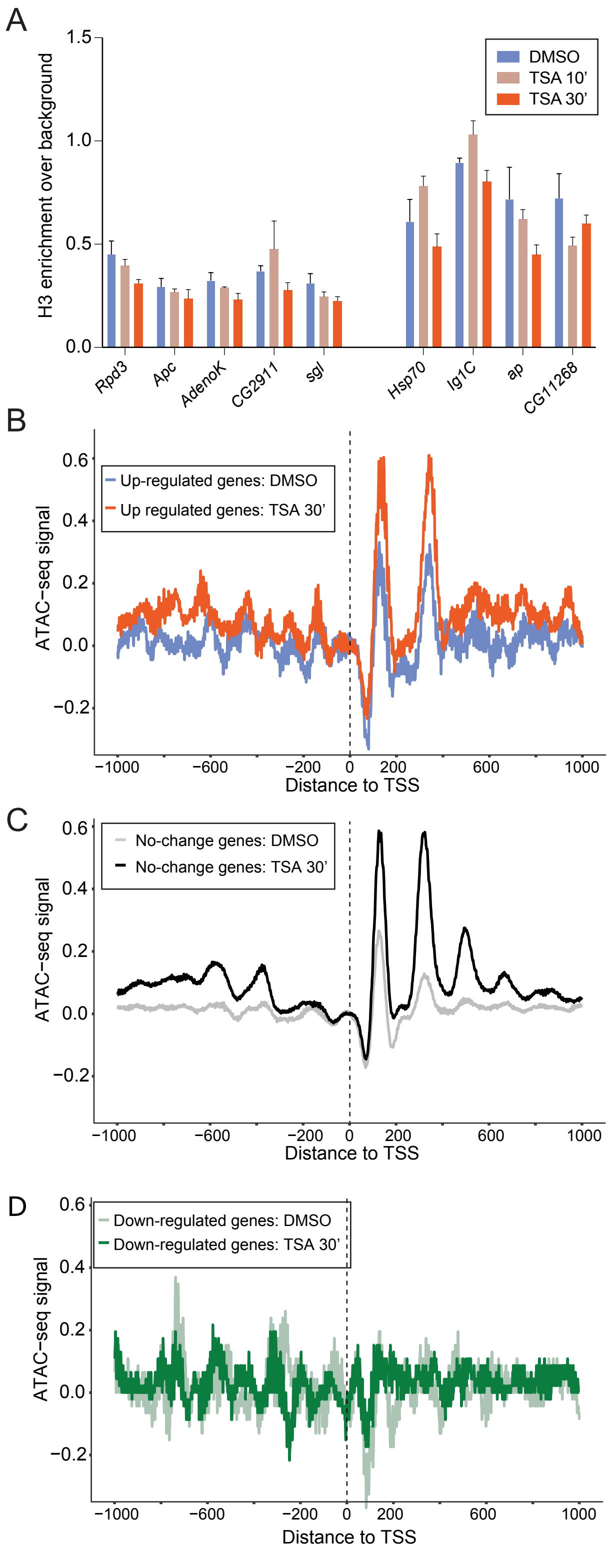
A
H3 enrichment over background
1.5
1.0
0.5
0.0
DMSO
TSA 10’
TSA 30’
Rpd3
Apc
AdenoK
CG2911
sgl
Hsp70
Ig1C
ap
CG11268
B
Up-regulated genes: DMSO
Up regulated genes: TSA 30’
ATAC−seq signal
0.6
0.4
0.2
0.0
−0.2
−1000
−600
−200
0
200
600
1000
Distance to TSS
C
No-change genes: DMSO
No-change genes: TSA 30’
ATAC−seq signal
Distance to TSS
D
Down-regulated genes: DMSO
Down-regulated genes: TSA 30’
ATAC−seq signal
Distance to TSS

Figure S5

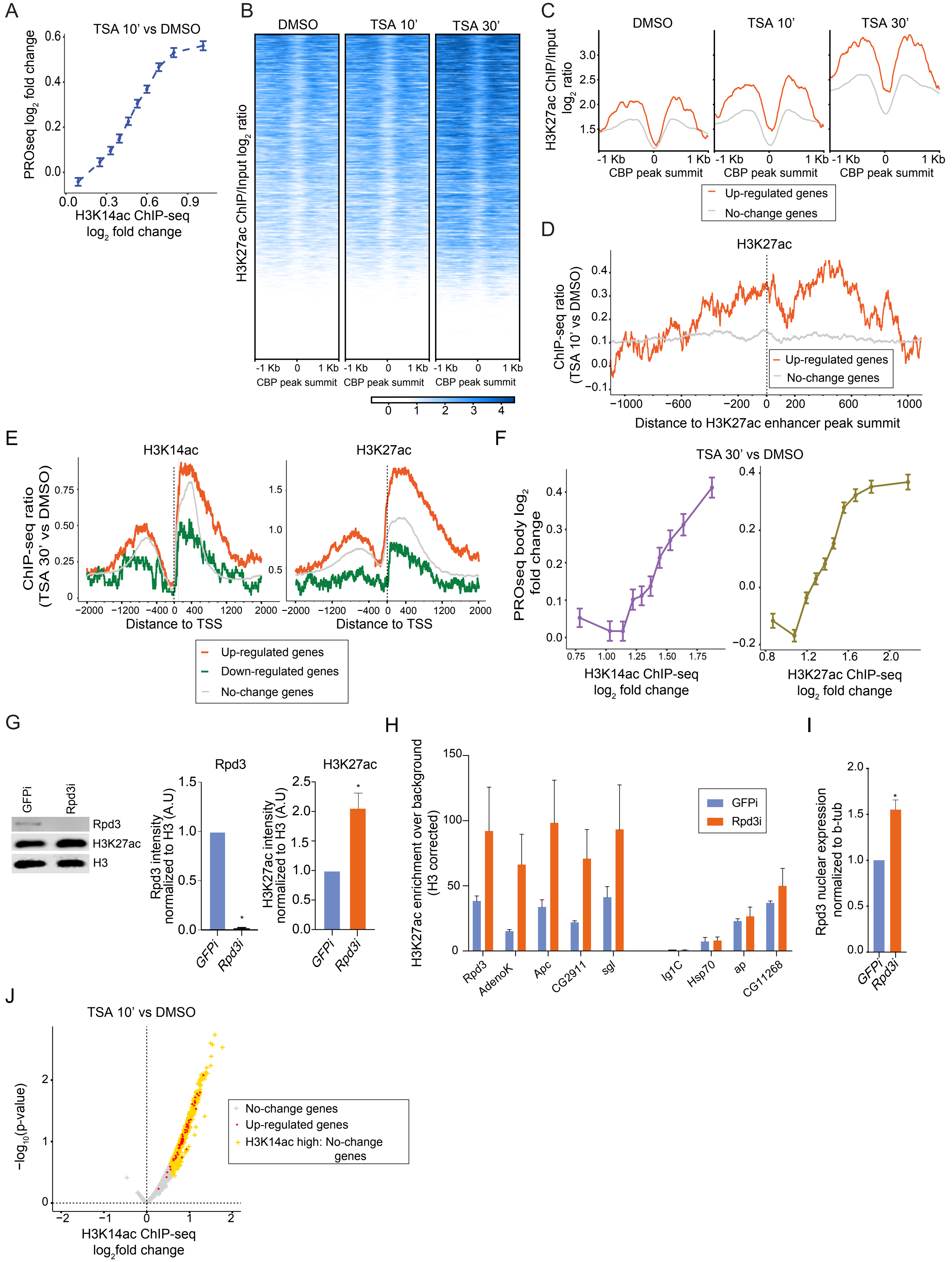
A
TSA 10' vs DMSO
PROseq log2 fold change
0.0
0.2
0.4
0.6
0.0
0.3
0.6
0.9
H3K14ac ChIP-seq
log2 fold change
B
DMSO
TSA 10'
TSA 30'
H3K27ac ChIP/Input log2 ratio
-1 Kb
0
1 Kb
CBP peak summit
0
1
2
3
4
C
DMSO
TSA 10'
TSA 30'
H3K27ac ChIP/Input
log2 ratio
1.5
2.0
2.5
3.0
-1 Kb
0
1 Kb
CBP peak summit
Up-regulated genes
No-change genes
D
H3K27ac
ChIP-seq ratio
(TSA 10' vs DMSO)
−0.1
0.0
0.1
0.2
0.3
0.4
−1000
−600
−200
0
200
600
1000
Distance to H3K27ac enhancer peak summit
Up-regulated genes
No-change genes
E
H3K14ac
H3K27ac
ChIP-seq ratio
(TSA 30' vs DMSO)
0
0.25
0.50
0.75
0.5
1.0
1.5
−2000
−1200
−400
0
400
1200
2000
Distance to TSS
Up-regulated genes
Down-regulated genes
No-change genes
F
TSA 30' vs DMSO
PROseq body log2
fold change
0.0
0.1
0.2
0.3
0.4
0.75
1.00
1.25
1.50
1.75
H3K14ac ChIP-seq
log2 fold change
−0.2
0.0
0.2
0.4
0.8
1.2
1.6
2.0
H3K27ac ChIP-seq
log2 fold change
G
GFPi
Rpd3i
Rpd3
H3K27ac
H3
Rpd3
Rpd3 intensity
normalized to H3 (A.U)
0.0
0.5
1.0
1.5
GFPi
Rpd3i
*
H3K27ac
H3K27ac intensity
normalized to H3 (A.U)
0.0
0.5
1.0
1.5
2.0
2.5
*
H
H3K27ac enrichment over background
(H3 corrected)
0
50
100
150
GFPi
Rpd3i
Rpd3
AdenoK
Apc
CG2911
sgl
lg1C
Hsp70
ap
CG11268
I
Rpd3 nuclear expression
normalized to b-tub
0.0
0.5
1.0
1.5
2.0
*
GFPi
Rpd3i
J
TSA 10' vs DMSO
−log10(p-value)
0
1
2
−2
−1
0
1
2
H3K14ac ChIP-seq
log2fold change
No-change genes
Up-regulated genes
H3K14ac high: No-change
genes

Figure S6

TSA 10’ vs DMSO

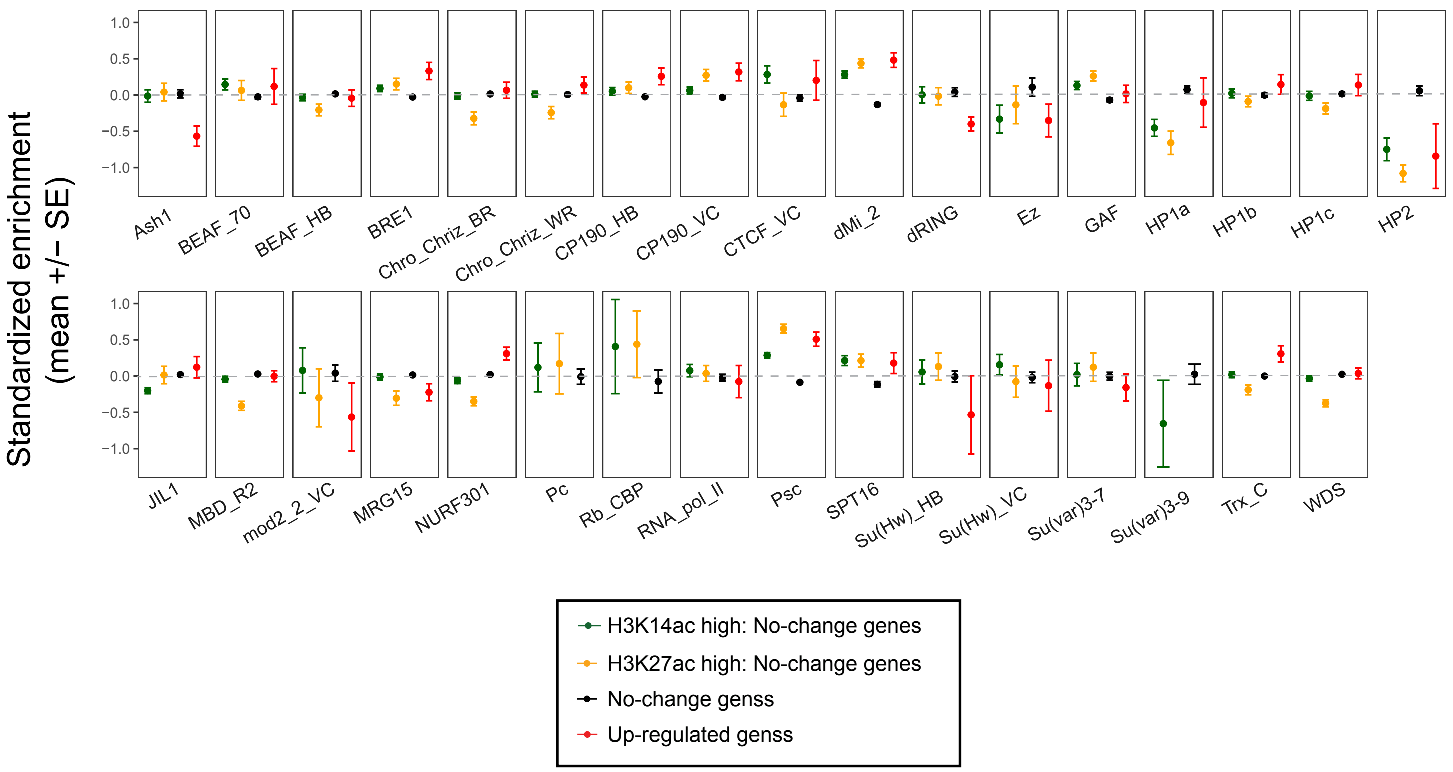

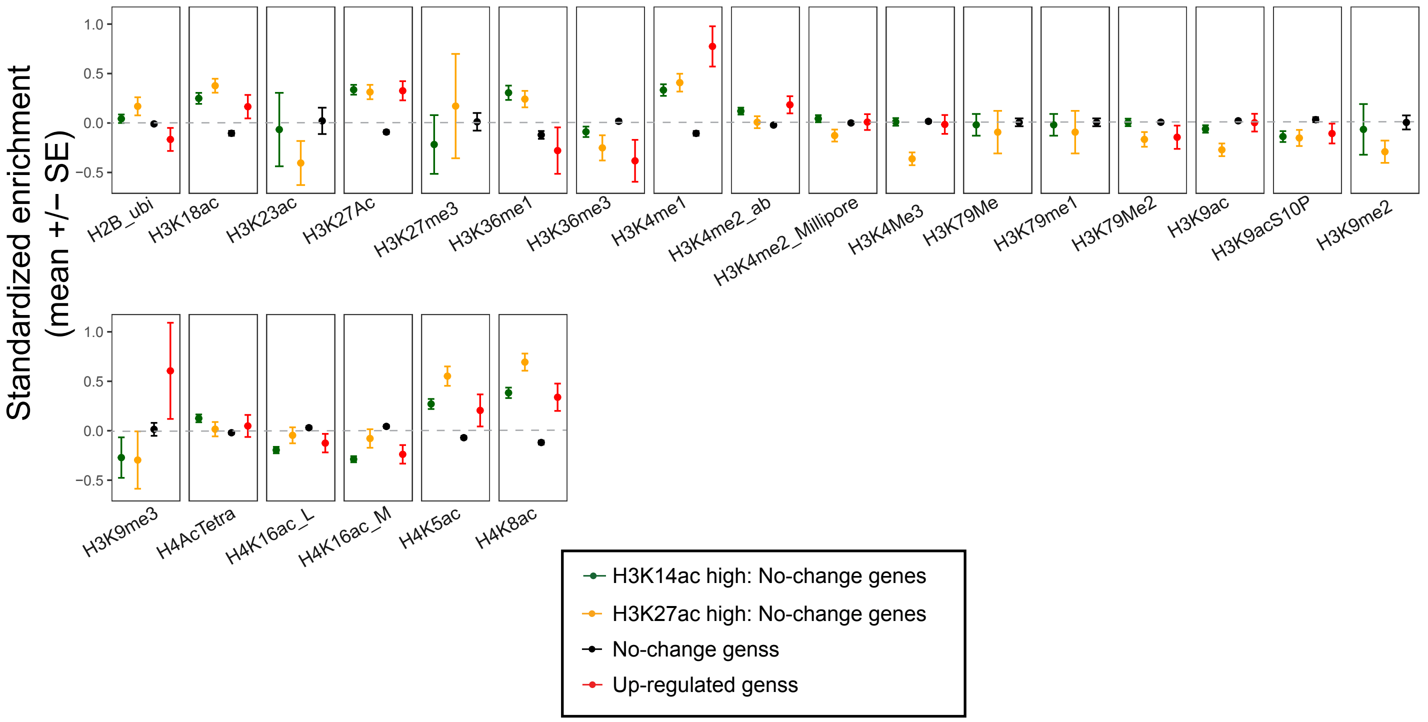

Figure S7

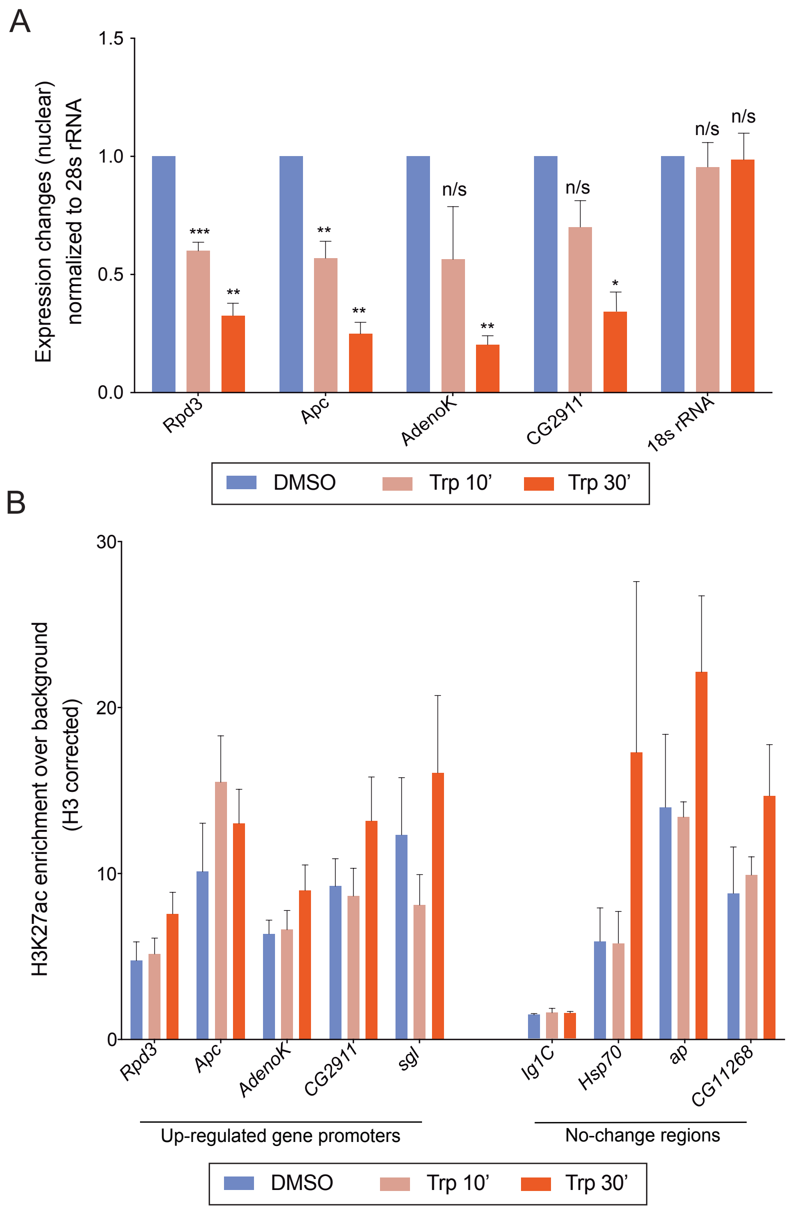
A
1.5
1.0
0.5
0.0
Expression changes (nuclear) normalized to 28s rRNA
***
**
**
**
n/s
**
n/s
*
n/s
n/s
Rpd3
Apc
AdenoK
CG2911
18s rRNA
DMSO
Trp 10'
Trp 30'
B
30
20
10
0
H3K27ac enrichment over background (H3 corrected)
Rpd3
Apc
AdenoK
CG2911
sgl
Ig1C
Hsp70
ap
CG11268
Up-regulated gene promoters
No-change regions
DMSO
Trp 10'
Trp 30'
