# Supplementary material for: Release of promoter–proximal paused Pol II in response to histone deacetylase inhibition
Source: Nucleic Acids Res. 2020 Apr 16;48(9):4877–90. doi: 10.1093/nar/gkaa234 (PMC7229826; doi:10.1093/nar/gkaa234)
Supplement: gkaa234_Supplemental_Files [file gkaa234_supplemental_files.zip › Table S2 (primers).pdf]

List of primers

ChIP-qPCR primers

Target regions

Gene	Gene feature	Forward primer sequence	Reverse primer sequence
Rpd3	Promoter	CAAAAAGCGCGTTTGTTACT	AACCCTAGCTGGCACTTTGT
Apc	Promoter	ACACAATTTCTCGGCAGCTT	GGCAATATCTGTGGCAAAC
AdenoK	Promoter	TCGTCATTCTCCTCGTGTGA	GCCACCTGAAGTGCAATACC
CG2911	Promoter	GACCCACTCGCTTTCAGAAC	AGTGCAAGGCGTGTGCTT
sgl	Promoter	GACATAACCAGCGCCAATG	AAACAAATCGAGCGACGAAT

Negative control primers

IG1C	Intergenic	AGCGCCGTGCGAAGGTAAAC	ATGGCCATTCCCCAAGGTCAT
Hsp70	Body	TGGGTGTCTACCAACATGGCAA	ATGAGGCGTTCCGAATCTGTGA
Ap	Intergenic	GCTGACCTCCCCCACTTAAT	TGCATCTGAAACTGGACCAA
CG11268	Promoter	TGCAGCTCTAGTCCTGATTCC	AAAAATGGACCCCAGAGGAC
Rpl32	Promoter	TTCACGATCTTGGGCCTGTATG	TTGTTGTGTCCTTCCAGCTTCA
Rpl32 5'	Intergenic	GGCACGGCGCCAAAATTAATCA	CCGATGCCACTGCCTCTTTGGT

Primers used for normalization

Norm_C1	Intergenic	TCGCTGATCAGATAAAGTGCTC	CAGGAATAAATGTAAAAGAATTTC GAG
Norm_C2	Intergenic	AAATCCAGTTGGCTTTGTGC	TTTGGGCTTCCAATTTGAGT

RT-qPCR primers - Drosophila

Target regions and control region primers

Gene	Gene feature	Forward primer sequence	Reverse primer sequence
Rpd3	Intron	CACAGCGCACTTTTTCCATA	CAACAAAAACACACAGAAGAGGA
Apc	Intron	TAATCATCGGCCAACATTCA	CGTGTGCCGCTAACTTTCTA
AdenoK	Intron	ATTCGCCCTGCTTATCAGTT	TATATATATCCGGCCAGGTTCA
CG2911	Exon/Intron	GCAACTCGGAACTTGCCTTA	AGTGCAAGGCGTGTGCTT
18s rRNA	-	ATTGGAGGGCAAGTCTGGTG	AATTTCACCTCTCGCGTCGT
28s rRNA	-	GCGGGGAAAGAAGACCCTTT	GCCCCAGTCAAACTCCCTAC
beta-tubulin	Exon	AAGCCTTGCGCCTGAACATAGC	TCCCGCCCCGTGGTCTG
GAPDH	Exon	GGATGGGGCCGAGATGATGA	TGGAGCCGAGTATGTGGTGGAGT

RT-qPCR primers - Human

Target regions and control region primers

Gene	Gene feature	Forward primer sequence	Reverse primer sequence
HDAC1_Hum	Intron	ACATTTTCTTGCCGAGAACC	TCTTCCCCCAGAAAAGATGA
p27_Hum	Intron	GCAACTGTGGTGGTCAGAAA	GAGAACAGGCCAGGACTGAG
p21_Hum	Intron	CGGTCTGTGAGTTTGAGCAG	CTTCACCAGCAAAGCACAAA
GAPDH_Hum	Exon	CCTGCTTCACCACCTTCTTGATGTC	CAAFGGTCATCCCAGAGCTGAACG
18s rRNA_Hum	-	GTAGCCCGTTGAACCCCATT	CCATCCAATCGGTAGTAGCC
ActB_Hum	Exon	GGACTTCGAGCAAGAGATGG	AGCACTGTGTTGGCGTACAG

Primers used for dsRNA synthesis

Rpd3_T7	Exon	TAATACGACTCACTATAGGG**GGAGGAGGCGTTCTATACCACCGATC**	TAATACGACTCACTATAGGG**GACTAATGTGCAGCTTAAAATCGG**
GFP_T7	Exon	GAATTAATACGACTCACTATAGGGAAGA**GGTGAGCAAGGGCGAGGAGCTGTTC**	GAATTAATACGACTCACTATAGGGAAGA**ATGCCGAGAGTGATCCCGGCGGCGG**
